# Inferring Gene Regulatory Networks From Single‐Cell RNA Sequencing Data by Dual‐Role Graph Contrastive Learning

**DOI:** 10.1002/advs.202518277

**Published:** 2025-11-29

**Authors:** Qiyuan Guan, Jiating Yu, Jieyi Pan, Fan Yuan, Jiadong Ji, Rusong Zhao, Zhi‐Ping Liu, Bingqiang Liu, Ling‐Yun Wu, Duanchen Sun

**Affiliations:** ^1^ School of Mathematics Shandong University Jinan 250100 China; ^2^ School of Mathematics and Statistics Nanjing University of Information Science & Technology Nanjing 210044 China; ^3^ School of Mathematics and Information Science Yantai University Yantai 264005 China; ^4^ Institute for Financial Studies Shandong University Jinan 250100 China; ^5^ Shandong Key Laboratory of Cancer Digital Medicine Jinan 250033 China; ^6^ State Key Laboratory of Reproductive Medicine and Offspring Health, Center for Clinical Reproductive Medicine, the First Affiliated Hospital of Nanjing Medical University, Center for Reproductive Medicine, Institute of Women, Children and Reproductive Health Shandong University Jinan 250012 China; ^7^ Department of Biomedical Engineering, School of Control Science and Engineering Shandong University Jinan Shandong 250061 China; ^8^ State Key Laboratory of Mathematical Sciences, Academy of Mathematics and Systems Science Chinese Academy of Sciences Beijing 100190 China; ^9^ School of Mathematical Sciences University of Chinese Academy of Sciences Beijing 100049 China

**Keywords:** gene regulatory network, graph contrastive learning, network inference, single‐cell RNA sequencing

## Abstract

Gene regulatory network (GRN) inference is fundamental to understanding the regulatory architecture underlying cellular processes. Accurate reconstruction of cell‐type‐specific GRNs is therefore essential for elucidating the mechanisms that govern cellular identity, development, and disease. However, inferring GRNs from single‐cell RNA sequencing data remains challenging due to data sparsity, noise, and the intrinsic complexity of gene regulation. Here, RegGAIN is presented, a novel deep learning‐based model designed to infer GRNs from single‐cell transcriptomic data. RegGAIN employs self‐supervised contrastive learning to maximize consistency of gene embeddings across perturbed graph views. To characterize regulatory directionality and capture the distinct regulator‐ and target‐driven patterns simultaneously, it leverages separate encoders to learn dual‐role representations for each gene. Comprehensive evaluations demonstrate that RegGAIN achieves accurate and robust GRN reconstruction, consistently outperforming existing methods. The biological relevance of the predicted regulatory interactions is further validated using external epigenetic data. Moreover, RegGAIN enables the discovery of GRN rewiring, revealing condition‐specific and temporally dynamic regulatory programs. Together, RegGAIN offers a powerful and generalizable framework for GRN inference, paving the way for deeper insights into cellular regulation across diverse biological contexts.

## Graphical abstract

RegGAIN is a novel and powerful deep learning framework for inferring gene regulatory networks (GRNs) from single‐cell RNA sequencing data. By integrating self‐supervised contrastive learning with dual‐role gene representations, it consistently outperforms existing methods in both accuracy and robustness. Moreover, RegGAIN effectively uncovers GRN rewiring, enabling the discovery of condition‐specific and dynamic regulatory programs across diverse biological contexts.

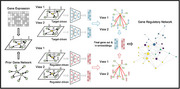

## 1 Introduction

Gene regulatory networks (GRNs) represent the intricate regulatory interactions between transcription factors (TFs) and their target genes, providing a systems‐level perspective of cellular regulation.^[^ ^1^ ^]^ Accurate GRN inference is essential for elucidating the mechanisms underlying disease pathogenesis, cellular differentiation, and other fundamental biological processes. Recent advances in single‐cell RNA sequencing (scRNA‐seq)^[^ ^2^ ^]^ have enabled the dissection of transcriptional heterogeneity at an unprecedented resolution, driving the development of GRN inference algorithms specifically tailored for single‐cell data. Existing methods span diverse computational paradigms, including correlation‐based approaches,^[^ ^3^ ^]^ tree‐based ensemble models,^[^ ^4^, ^5^ ^]^ Gaussian graphical models,^[^ ^6^ ^]^ and deep learning‐based frameworks.^[^ ^7^, ^8^, ^9^, ^10^ ^]^

In parallel, the rapid expansion of large‐scale biological databases presents new opportunities to incorporate prior knowledge into GRN inference.^[^ ^11^ ^]^ Resources such as gene interaction networks and TF–target annotations offer valuable regulatory evidence derived from experimental and computational studies.^[^ ^12^, ^13^, ^14^, ^15^ ^]^ However, a significant challenge is to reconcile the broad, context‐independent prior knowledge with the high‐resolution, context‐specific information captured by scRNA‐seq data. Since prior knowledge is typically represented as network structures, graph‐based models provide a promising framework to leverage the topological and contextual information embedded in GRNs. In particular, graph neural networks (GNNs) have shown strong potential in this setting.^[^ ^16^ ^]^ Among them, graph contrastive learning has emerged as a powerful paradigm for graph representation learning,^[^ ^17^, ^18^ ^]^ achieving superior performance by learning discriminative embeddings through self‐supervised training. For instance, CEFCON^[^ ^19^ ^]^ integrates deep graph infomax^[^ ^20^ ^]^ with graph attention networks to model regulatory context and assign confidence scores to TF–target interactions.

Despite these advances, the unique characteristics of GRNs pose challenges for general‐purpose contrastive learning frameworks. Standard graph augmentation strategies often fail to generate sufficiently distinct views for GRNs, as the inherent resilience of their scale‐free topology to random alterations provides a weak supervisory signal for contrastive learning. Furthermore, conventional GNN encoders are predominantly designed for undirected graphs and cannot capture regulatory directionality, thereby overlooking the inherently asymmetric nature of TF–target gene interactions. These limitations highlight the need for specialized contrastive learning frameworks that explicitly model both the directional topology and regulatory semantics of GRNs, while effectively incorporating prior knowledge to improve inference accuracy from scRNA‐seq data.

To address these challenges, we present RegGAIN, a self‐supervised graph contrastive learning framework for GRN inference from scRNA‐seq data. RegGAIN integrates species‐specific prior regulatory networks and introduces graph perturbations that preserve key topological properties while generating informative augmented views for contrastive learning. It further employs separate encoders to learn dual‐role representations, simultaneously characterizing each gene's regulator‐driven and target‐driven patterns to enable direction‐aware GRN reconstruction. Through comprehensive evaluations on benchmark datasets and external epigenomic resources, RegGAIN achieves accurate and robust GRN reconstruction, consistently outperforming existing methods. Additionally, RegGAIN enables biological discovery: in multiple myeloma, it identified dysregulated TFs underlying condition‐specific network rewiring, while in a time‐series data, it revealed developmentally relevant regulons and characterized TF interaction landscapes. Together, RegGAIN offers a powerful and generalizable framework for GRN inference, paving the way for deeper insights into cellular regulation across diverse biological contexts.

## 2 Results

### 2.1 Overview of RegGAIN

**Figure 1:**
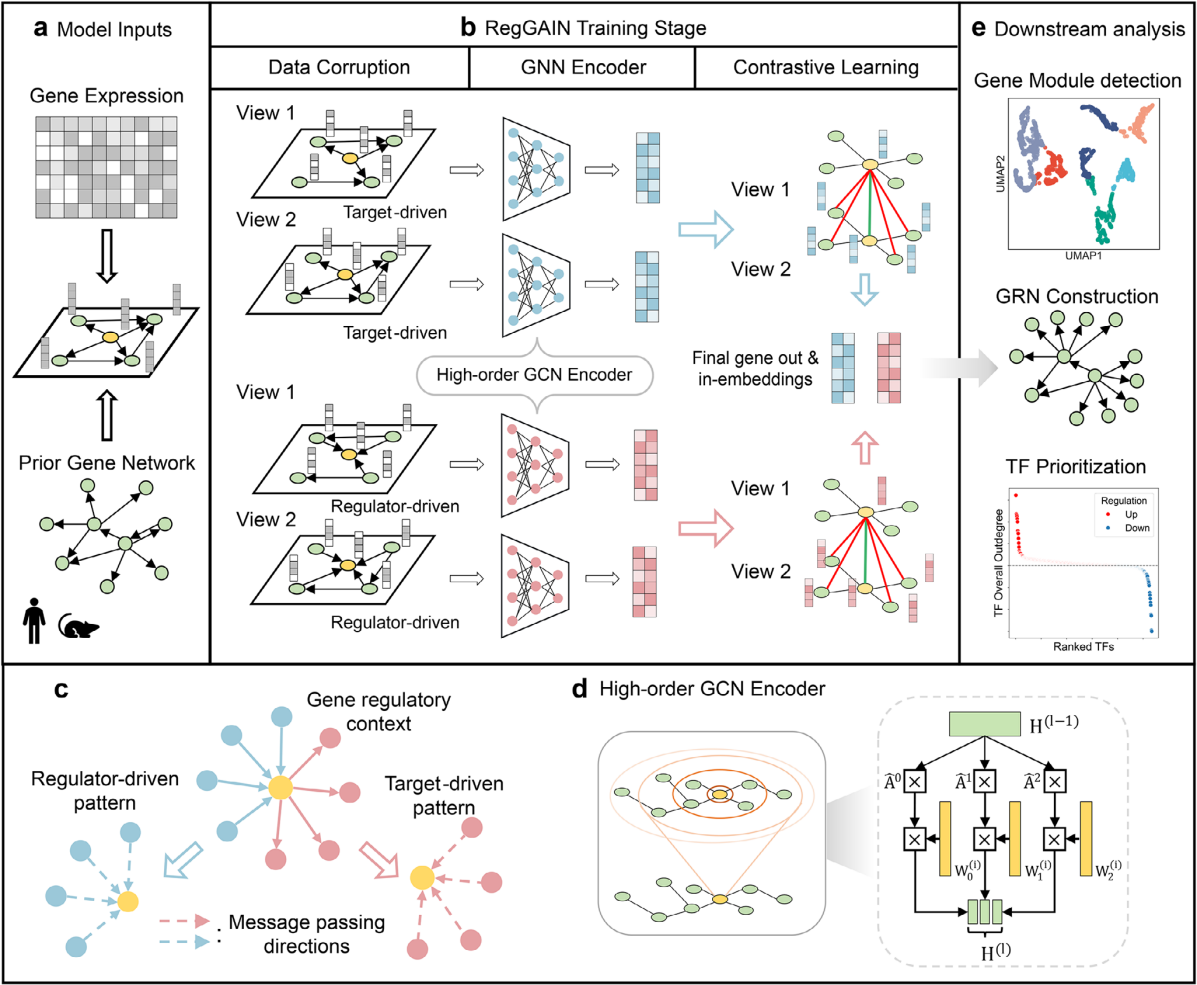
Overview of RegGAIN. a) RegGAIN integrates a gene expression matrix with a prior gene network derived from existing biological knowledge as inputs. b) During training, RegGAIN first generates two graph views via random (View 1) and topology‐aware (View 2) structural and feature corruption. To capture the distinct regulator‐driven and target‐driven patterns, it processes each view using a dual high‐order GCN encoder. Contrastive learning then aligns node representations across the views to enhance regulatory signals. c) Regulator‐ and target‐driven patterns capture gene representations in their regulatory contexts, reflecting whether they act as regulators or targets. These patterns aggregate information from neighboring genes in upstream or downstream directions. d) The high‐order GCN encoder simultaneously aggregates features from different neighborhood orders (0‐hop, 1‐hop, and 2‐hop), each with its own trainable weights, and concatenates them to capture both direct and indirect dependencies. e) RegGAIN outputs a refined GRN along with low‐dimensional embeddings, enabling downstream analyses such as detecting gene regulatory modules and prioritizing TFs across different conditions during disease progression.

RegGAIN is a novel deep learning‐based model designed to infer GRNs from single‐cell transcriptomic data. It takes as input a normalized single‐cell gene expression profile of a specific cell type or state, and a species‐specific prior gene interaction network (**Figure** **1**a). RegGAIN employs self‐supervised contrastive learning by generating two perturbed graph views of the prior network through biologically informed structural and feature corruption (Figure 1b). It then maximizes the consistency of gene embeddings across views while distinguishing them from embeddings of other genes. To characterize regulatory directionality and capture the distinct regulator‐ and target‐driven patterns (Figure 1c), RegGAIN utilizes separate encoders to learn dual‐role representations for each gene. Besides, a high‐order graph convolutional network (GCN) encoder is incorporated to model long‐range regulatory dependencies (Figure 1d). Finally, by quantifying the similarity between gene out‐ and in‐embeddings, RegGAIN outputs directed GRNs, where edges represent predicted TF–target regulatory strengths derived from the learned embeddings. These reconstructed GRNs enable downstream analyses such as gene module detection, network rewiring under varying conditions (e.g., disease versus control or across time points), and TF prioritization for biological or clinical interpretation (Figure 1e).

### 2.2 RegGAIN Achieves Accurate and Robust GRN Reconstruction

To evaluate the performance of RegGAIN in reconstructing GRNs, we employed the BEELINE benchmarking framework,^[^ ^21^ ^]^ which includes three categories of ground‐truth networks and seven diverse scRNA‐seq datasets (Note S1, Supporting Information). For each dataset, we selected highly variable TFs combined with the top *N* most variable genes (*N* = 500 and 1000), ensuring the inclusion of biologically informative features (Tables S1 and S2, Supporting Information). We benchmarked RegGAIN against several state‐of‐the‐art GRN inference algorithms, including GRNBoost2,^[^ ^5^ ^]^ DeepSEM,^[^ ^8^ ^]^ KEGNI,^[^ ^22^ ^]^ NetREX,^[^ ^23^ ^]^ and CEFCON.^[^ ^19^ ^]^ Quantitative evaluations were conducted using two primary metrics: the Area Under the Precision‐Recall Curve ratio (AUPRC ratio) and the Early Precision Ratio (EPR) (Experimental Section).

**Figure 2:**
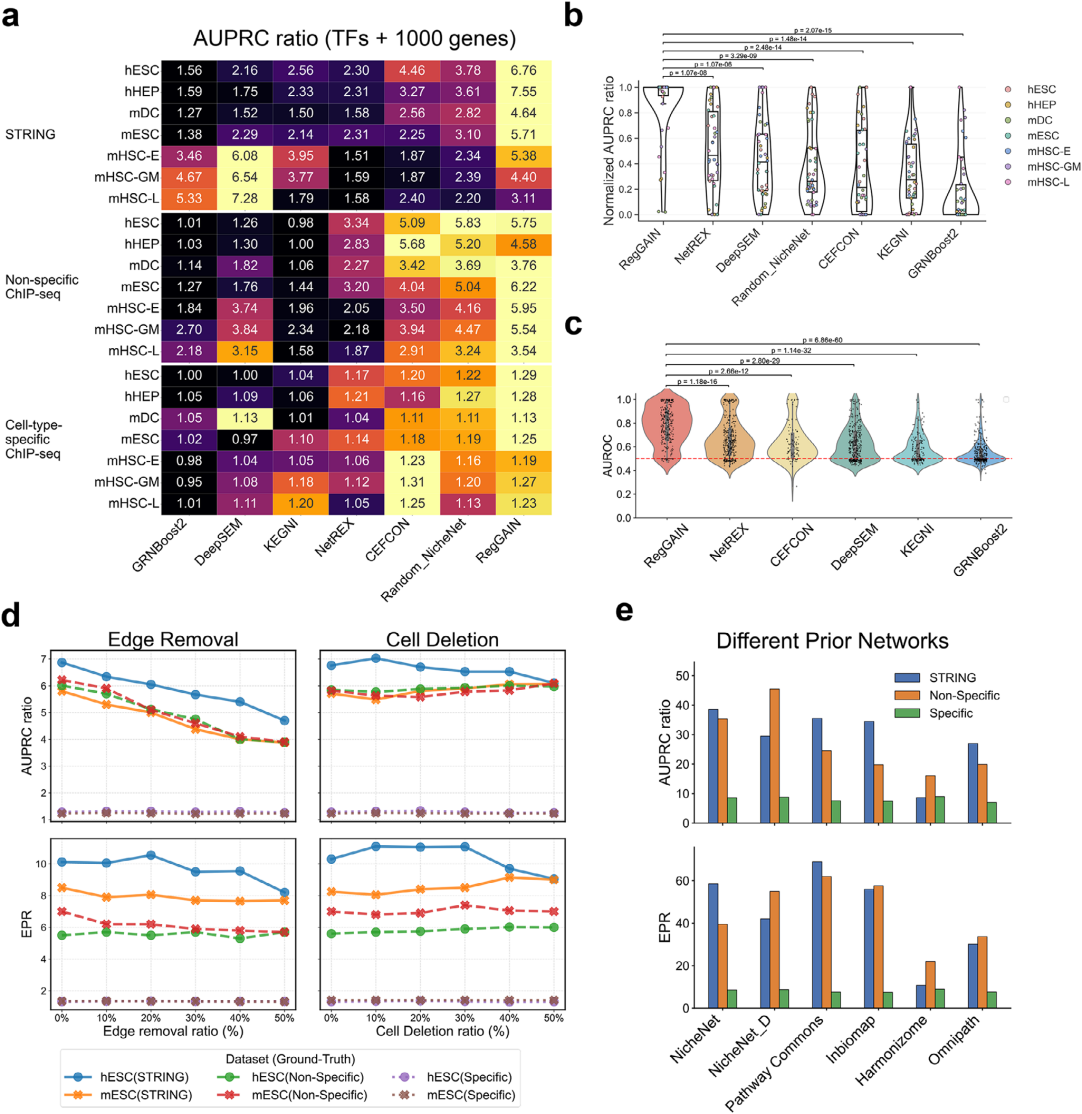
RegGAIN achieves accurate and robust GRN reconstruction. a) Heatmap shows the AUPRC ratio of GRN inference on seven scRNA‐seq datasets, evaluated against three distinct ground‐truth sources, using the 1000 most variable genes and all variable TFs. b) Violin plots show the distributions of normalized AUPRC ratio across different ground‐truth networks and datasets. Statistical significance was evaluated using the Wilcoxon rank‐sum test, comparing the performance of RegGAIN with that of each other method. c) Violin plots illustrate the distributions of AUROC achieved by each model for predicting the targets of individual transcription factor, evaluated using the STRING ground‐truth network. Statistical significance was evaluated using the Wilcoxon rank‐sum test, comparing the performance of RegGAIN with that of other method. d) Robustness of RegGAIN under perturbations, assessed by progressively removing edges from the prior network (left) or deleting cells from the scRNA‐seq data (right) using TF+1000 HVGs on hESC and mESC datasets with three ground‐truth sources. e) Overall performance of RegGAIN (Top: AUPRC ratio; Bottom: EPR) across seven datasets using six different prior networks.

Across 42 benchmarking scenarios spanning diverse datasets, prior networks, and evaluation metrics, RegGAIN consistently exhibited superior overall performance, ranking first in 67% of the cases based on both AUPRC ratio and EPR (**Figure** **2**a; Figures S1a and S2a, Supporting Information), and placing within the top three performance in 95% of all evaluations. Specifically, compared to methods that rely solely on gene expression, such as GRNBoost2 and DeepSEM, RegGAIN achieved substantially higher performance, with AUPRC ratios improved by 117% and 57% over GRNBoost2 and DeepSEM, and EPR scores increased by 92% and 45%, respectively. Prior‐informed methods, such as NetREX and CEFCON, performed well under the EPR metric in certain human cell types but lacked stability, whereas RegGAIN demonstrated consistently high GRN reconstruction accuracy across diverse datasets and networks. Furthermore, RegGAIN exceeded Random_NicheNet, which randomly samples edges from the same prior network, confirming that its superior performance arises from both the informative prior and its self‐supervised, direction‐aware architecture. RegGAIN's superior performance is further underscored when the benchmarking results are viewed in an aggregated manner and when evaluating its accuracy in predicting the targets of individual transcription factors (Figure 2b,c; Figure S1b,c, Supporting Information).

While comprehensive priors like NicheNet can help mitigate false positives and, in some cases, outperform methods that rely solely on scRNA‐seq data, their scope remains inherently generic. RegGAIN addresses this limitation by incorporating prior knowledge without being exclusively reliant on them. Through integration with scRNA‐seq data, RegGAIN prunes irrelevant or redundant edges from broad prior networks, producing cell‐type‐specific, data‐driven GRNs that are both refined and contextually accurate (Tables S3 and S4, Supporting Information). Importantly, this refinement prevents generic topological features of the prior, such as hub nodes, from being indiscriminately propagated into the inferred network (Figure S3, Supporting Information). Next, to assess robustness, we conducted two perturbation analyses on the human and mouse embryonic stem cells (hESC and mESC) datasets: random removal of prior network edges and downsampling of cells by up to 50% (Experimental Section). RegGAIN maintained high AUPRC ratio and EPR across all perturbation levels, demonstrating strong robustness to both incomplete prior knowledge and reduced sample sizes (Figure 2d; Figure S2b, Supporting Information). Notably, even with 50% of the prior edges removed, the AUPRC ratio and EPR decreased by only ≈27% and 10%, respectively. In contrast, performance of RegGAIN remained stable under cell downsampling, with both evaluation metrics largely unaffected. Furthermore, RegGAIN proved highly resilient to noisy priors, demonstrating a strong ability to correct for a substantial number of erroneous edges while maintaining robust performance (Experimental Section, Figures S4–S6, Supporting Information).

To further examine the generalizability of RegGAIN, we evaluated its GRN reconstruction performance using prior networks from diverse sources, including a directionally filtered version of NicheNet (NicheNet_D), a high‐confidence protein–protein interaction network (InBioMap),^[^ ^24^ ^]^ a meta‐database of curated biological pathways (Pathway Commons),^[^ ^25^ ^]^ an integrated source of functional associations (Harmonizome),^[^ ^15^ ^]^ and a comprehensive meta‐database of molecular interactions (OmniPath) (Note S2, Supporting Information).^[^ ^14^ ^]^ We observed that different prior networks exhibited distinct advantages under specific validation settings (Figure 2e; Figure S2c and Table S5, Supporting Information). For example, NicheNet_D performed well under non‐cell‐type‐specific benchmarks, whereas Harmonizome showed superior performance in cell‐type‐specific contexts. These results indicated that coupling RegGAIN with appropriate priors enables flexible adaptation to diverse analytical scenarios. Overall, we recommend NicheNet as a default prior due to its consistently stable and well‐balanced performance across varied evaluation settings.

### 2.3 Dual‐Role Representations and Architectural Components Jointly Enable RegGAIN to Capture Regulatory Programs

**Figure 3:**
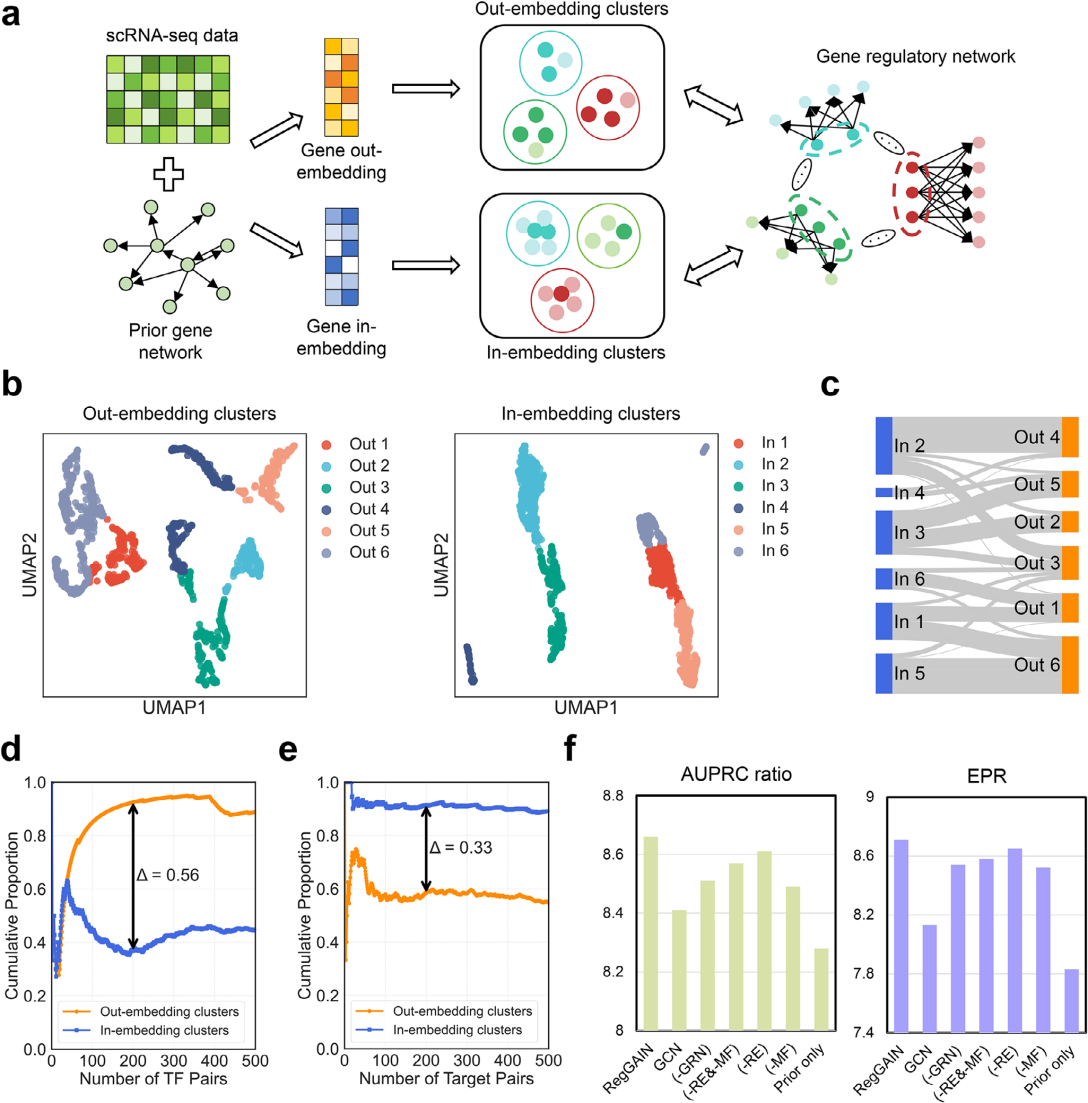
Dual‐role representations and architectural components jointly enable RegGAIN to capture regulatory programs. a) Schematic of the RegGAIN pipeline for generating out‐embedding clusters and in‐embedding clusters. b) UMAP visualization of gene out‐embeddings (left) and gene in‐embeddings (right) for the hESC dataset, with clustering performed using the K‐means algorithm (num_clusters = 6). c) Sankey diagram illustrating the correspondence between out‐embedding clusters and in‐embedding clusters. d,e) Line plots showing the cumulative proportion of the most similar (d) TF pairs and (e) target gene pairs that fall within the same cluster. f) Comparison of RegGAIN with its ablated versions, evaluated by AUPRC ratio (left) and EPR (right), using cell‐type‐specific ground‐truth networks across seven scRNA‐seq datasets.

To model the multifaceted nature of transcriptional regulation, RegGAIN learns distinct feature representations for each gene by explicitly separating its roles as a regulator (out‐embedding) and as a target (in‐embedding) within the GRN. In this section, to validate this design, we analyzed these embeddings to test whether the out‐embeddings capture regulator‐driven patterns, while the in‐embeddings encode target‐driven patterns (**Figure** **3**a).

Using the hESC dataset, we applied k‐means clustering separately to the gene out‐ and in‐embeddings (Figure 3b). The resulting clusters revealed clear distinctions between the two embedding spaces (Figure 3c). Specifically, two out of six out‐embedding clusters were significantly enriched for TFs (Fisher's exact test *p* = 4.1e‐51 and 4.8e‐7, respectively), supporting the conclusion that out‐embeddings capture shared regulatory functions. In contrast, these TFs were scattered across multiple in‐embedding clusters, suggesting that the absence of similar structural patterns in the in‐embedding space (e.g., genes from out‐embedding cluster 3 were distributed across in‐embedding clusters 1, 2, 3, 5, and 6).

To evaluate whether the embedding‐derived clusters reflect biologically meaningful regulatory patterns, we calculated the pairwise Jaccard similarity between TFs based on their predicted downstream target genes from the GRN (Experimental Section). Ranking all TF pairs by similarity, we observed that top‐ranking TFs were more likely to co‐occur in the same out‐embedding clusters than in the in‐embedding clusters. Notably, among the top 200 similar TF pairs, out‐embedding clusters exhibited a 56% improvement in cumulative proportion compared to in‐embedding clusters (Figure 3d). In contrast, for target gene pairs, in‐embedding clusters showed a 33% higher cumulative proportion compared to out‐embedding clusters (Figure 3e). Similar results were also obtained using the Leiden algorithm at varying resolutions as well as on an independent mESC dataset (Figures S9–S12, Supporting Information). These observations further validate that RegGAIN's dual‐role representations effectively capture distinct identities underlying transcriptional regulation.

These analyses confirm that the dual‐role embeddings capture distinct aspects of transcriptional regulation, consistent with our design. Moreover, RegGAIN's architecture comprises multiple components beyond the embedding scheme. To disentangle their individual contributions to overall performance, we performed a series of ablation experiments. Specifically, we i) replaced the high‐order message aggregation GCN with a standard GCN, ii) removed the power‐law‐aware perturbation, iii) disabled the graph perturbation, iv) disabled edge removal during the graph perturbation, v) disabled feature masking during the graph perturbation, and vi) relied solely on the prior gene network. Model performance was evaluated across seven datasets using cell‐type‐specific ground‐truth regulatory networks, providing biologically relevant benchmarks for GRN inference. As a result, each component was found to contribute to RegGAIN's superior performance in both AUPRC ratio and EPR (Figure 3f), highlighting the importance of the model's full architecture for accurately capturing context‐specific gene regulatory interactions.

### 2.4 Validating GRN Prediction using External Epigenetic Data

Chromatin accessibility is a key epigenetic feature that reflects the openness of genomic regions and influences transcriptional activity by modulating the binding of TFs to cis‐regulatory elements.^[^ ^26^ ^]^ Previous studies have demonstrated that combining TF binding motifs with chromatin accessibility profiles facilitates the identification of cell‐type‐specific functional TF binding sites.^[^ ^27^ ^]^ Building on this principle, we examined whether the binding motifs of RegGAIN‐predicted regulators are enriched in accessible chromatin regions near the transcription start site (TSS) of their target genes.

**Figure 4:**
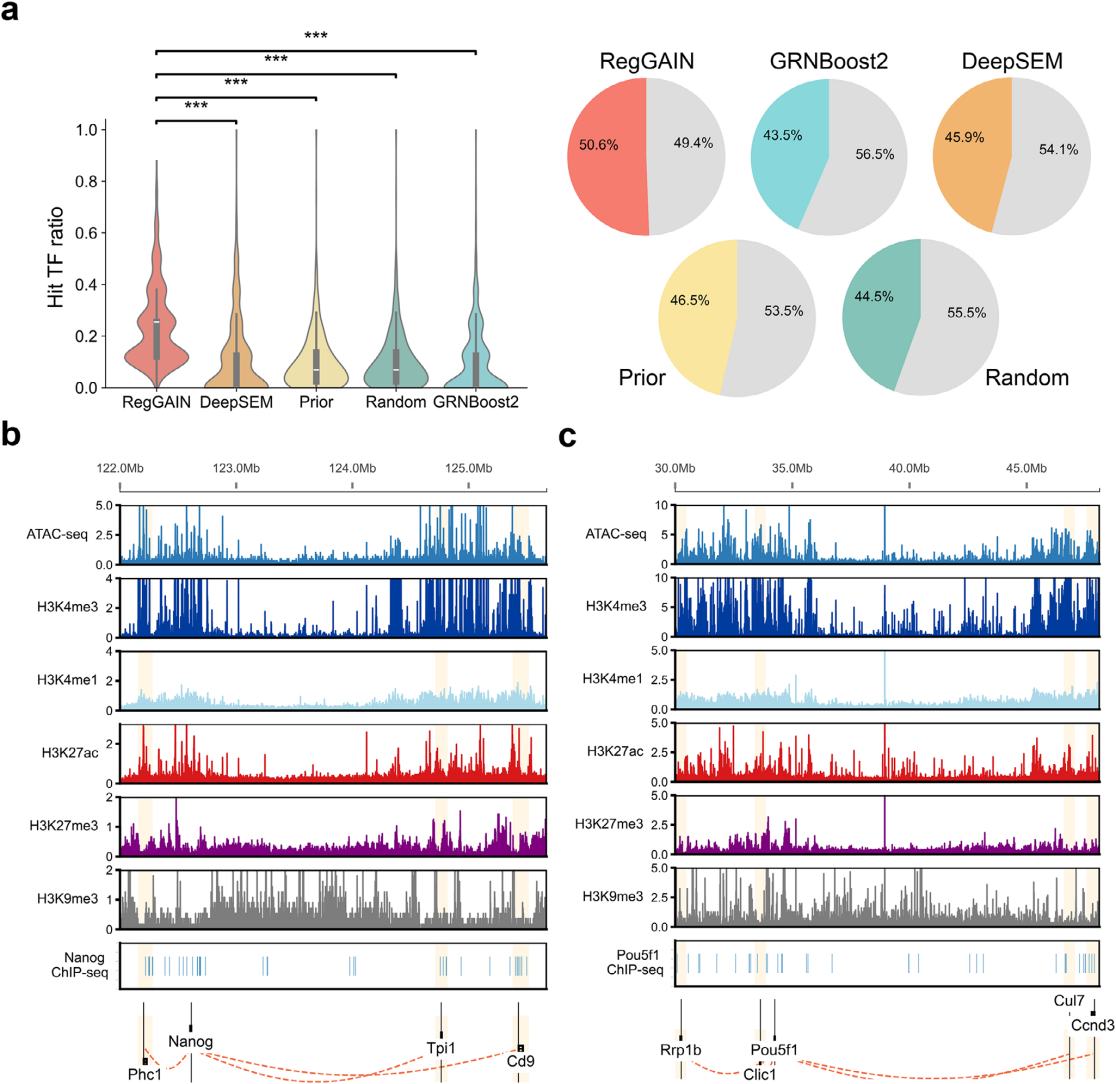
Validating GRN prediction using external epigenetic data. a) Violin plots and pie charts show the distribution of TF hit ratios across each target gene and the proportion of target genes with at least one predicted TF regulator from RegGAIN, DeepSEM, GRNBoost2, prior network, and a random baseline. Statistical significance was evaluated using the Wilcoxon rank‐sum test (***p < 0.001). b,c) Genome browser tracks show representative examples of epigenetic signals (ATAC‐seq, histone modifications, and ChIP‐seq) supporting RegGAIN‐predicted regulatory interactions for (b) Nanog and (c) Pou5f1.

To evaluate the biological relevance of RegGAIN's predicted regulatory interactions, we inferred a GRN from mESC scRNA‐seq data and validated the predicted TF–target gene pairs using single‐cell Assay for Transposase‐Accessible Chromatin using Sequencing (scATAC‐seq) data from the same cell type. For each target gene, scATAC‐seq peaks within ±200 kb of the TSS were annotated as candidate regulatory elements. We then quantified the fraction of predicted regulators whose binding motifs overlapped with these accessible regions (Experimental Section). Compared to two alternative strategies (randomly selected TFs and TFs from prior networks) and two other GRN inference methods, RegGAIN's predictions showed substantially higher motif enrichment within accessible chromatin regions, highlighting the accuracy of the GRN reconstructed by RegGAIN from an epigenetic perspective (**Figure** **4**a).

We further assessed the epigenetic consistency of RegGAIN‐inferred regulatory interactions by integrating scATAC‐seq,^[^ ^28^ ^]^ Chromatin Immunoprecipitation Sequencing (ChIP‐seq),^[^ ^29^ ^]^ and histone modification profiles.^[^ ^30^ ^]^ The analysis focused on histone marks indicative of active regulatory regions (H3K4me3, H3K4me1, and H3K27ac) as well as those associated with repressive chromatin states (H3K27me3 and H3K9me3).^[^ ^31^ ^]^ As a case study, we examined top‐scoring cis‐regulatory predictions for Nanog and Pou5f1, two core TFs that are essential for establishing and maintaining embryonic stem cell pluripotency.^[^ ^32^, ^33^ ^]^ For Nanog, RegGAIN identified *Phc1, Tpi1*, and *Cd9* as high‐confidence targets. The genomic loci of these predicted targets exhibited clear epigenetic hallmarks of active regulation, including chromatin accessibility and strong enrichment of H3K4me3 and H3K27ac (Figure 4b). Notably, these features directly overlapped with Nanog binding sites confirmed by ChIP‐seq, providing physical validation for the predicted interactions. Similarly, for Pou5f1, top‐predicted cis‐targets such as *Rrp1b* and *Clic1* also displayed accessible chromatin and active histone marks that coincided precisely with robust Pou5f1 ChIP‐seq peaks (Figure 4c).

Collectively, these pieces of evidence demonstrated that RegGAIN accurately reconstructed GRNs in agreement with external epigenetic data. The predicted interactions align with accessible chromatin and TF binding motifs, and further coincided with active histone marks and ChIP‐seq‐validated binding events, providing additional support for the biological relevance of RegGAIN's predicted regulatory interactions.

### 2.5 RegGAIN Uncovers GRN Rewiring in Multiple Myeloma

Multiple myeloma (MM) is a hematological malignancy characterized by the clonal proliferation of plasma cells in the bone marrow, often resulting in poor clinical outcomes.^[^ ^34^, ^35^ ^]^ To dissect the transcriptional regulatory alterations underlying MM pathogenesis, we applied RegGAIN to scRNA‐seq data from MM patients and normal bone marrow (NBM) donors,^[^ ^36^ ^]^ inferring GRNs at the individual sample level to explore regulatory rewiring between malignant and healthy states (Experimental Section).

**Figure 5:**
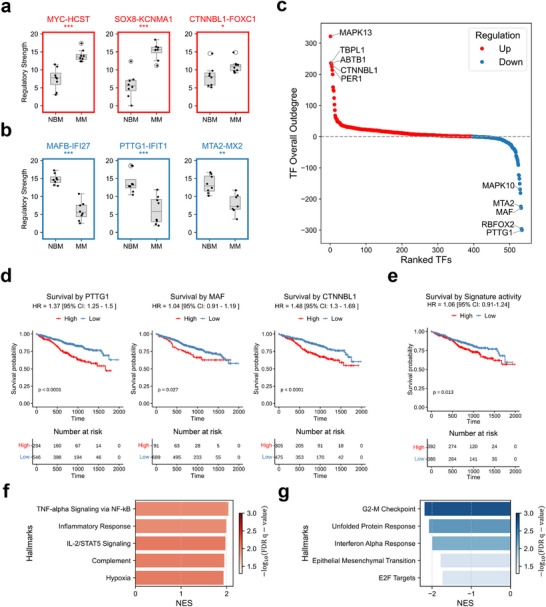
RegGAIN uncovers GRN rewiring in multiple myeloma. a) Representative examples of TF–target pairs with significantly increased regulatory strength in MM compared to NBM. b) Representative examples of TF–target pairs with significantly decreased regulatory strength in MM. c) Global ranking of TFs by differential overall out‐degree (MM versus NBM). Red and blue dots represent TFs with predominantly up‐ or downregulated regulatory edges, respectively; light‐colored dots indicate mixed or unchanged activity. Selected top TFs are labeled. d) Kaplan–Meier survival curves illustrate the clinical relevance of the selected TFs on the independent multiple myeloma cohort. Tick marks indicate censoring events. The statistical *p*‐values were determined by the two‐tailed log rank‐sum test. e) Kaplan–Meier survival curves illustrate the clinical relevance of the MM signature. f) Significantly enriched hallmark pathways among target genes of upregulated TF–target regulations in MM, ranked by normalized enrichment score (NES). NES and adjusted *p*‐values were computed using the GSEA test. g) Significantly enriched hallmark pathways among target genes of downregulated TF–target regulations in MM, ranked by NES.

As a result, numerous regulatory interactions exhibited significantly altered strength, with several oncogenesis‐related interactions markedly upregulated in MM (**Figure** **5**a). For instance, the *MYC*–*HCST* link was enhanced; *HCST*, an immunoreceptor signaling adapter,^[^ ^37^ ^]^ appears to be increasingly regulated by the master oncogene *MYC*, suggesting a mechanism that couples proliferative signaling with immune microenvironment modulation.^[^ ^38^, ^39^ ^]^ We also observed heightened activity in circuits involving developmental TFs co‐opted for malignancy. A notable example is the enhanced regulation of the potassium channel *KCNMA1* by *SOX8*. Since *KCNMA1* dysregulation is an emerging cancer hallmark^[^ ^40^ ^]^ and *SOX8* promotes proliferation in other cancers, this interaction likely confers a pro‐survival electrophysiology shift in MM.^[^ ^41^ ^]^ In contrast, several immune‐related regulatory circuits were significantly downregulated, particularly those involving interferon‐stimulated genes (ISGs) (Figure 5b).^[^ ^42^ ^]^ For example, the diminished regulation of *IFI27* by *MAFB*, which is essential for normal plasma cell differentiation, implies that MM cells suppress antiviral immunity by disrupting native differentiation programs.^[^ ^43^ ^]^ Similarly, reduced control of the *IFIT1* and *MX2* by the oncogene *PTTG1*, and the chromatin remodeler *MTA2*, respectively, underscores a broader strategy of immune evasion through selective decoupling of core regulatory mechanisms.

To identify master TFs driving regulatory rewiring in MM, we ranked them by their differential out‐degree (Experimental Section), revealing distinct sets with predominantly increased or decreased regulatory activity (Figure 5c). In an independent patient cohort, top‐rewired TFs showed strong clinical relevance. For example, both *PTTG1* and *MTA2* were significantly associated with poor survival (*PTTG1*: HR = 1.37, *p* < 0.0001; *MTA2*: HR = 1.28, *p* = 0.0011; Figure 5d; Figure S13, Supporting Information), consistent with their known oncogenic and repressive functions.^[^ ^44^, ^45^ ^]^ Interestingly, despite *MAF* being a well‐established MM oncogene,^[^ ^46^ ^]^ its regulatory out‐degree was reduced, suggesting a refocused program targeting a core set of tumor‐promoting targets. Our analysis also identified *CTNNBL1*, a top TF with increased regulatory activity that has not been extensively studied in MM, but is involved in spliceosome function and β‐catenin regulation.^[^ ^47^ ^]^ Its strong association with poor survival (HR = 1.48, *p* < 0.0001) suggests a previously unrecognized role in MM progression (Figure 5d). Furthermore, we constructed an MM signature based on the top upregulated and downregulated genes (Experimental Section). Patients with higher signature scores exhibited significantly worse survival time compared to those with lower scores (Figure 5e). This observation indicated that the MM signature was associated with worse survival and could provide potential drug targets for further investigation.

Finally, we performed Gene Set Enrichment Analysis (GSEA)^[^ ^48^ ^]^ to investigate the functions of regulatory reprogramming in MM. We found that upregulated regulons were significantly enriched for hallmark pathways such as TNF‐α signaling via NF‐κB, inflammatory response, and hypoxia—key drivers of cell proliferation, survival, and adaptation to the bone marrow microenvironment (Figure 5f). In contrast, pathways associated with anti‐tumor functions were significantly suppressed. The downregulation of Interferon alpha response aligned with reduced ISG regulation, reinforcing a strategy of immune evasion (Figure 5g). Furthermore, repression of G2‐M Checkpoint, E2F Targets and the unfolded protein response suggests disrupted cell cycle control and enhanced tolerance to proteotoxic stress—both critical features of MM pathophysiology.^[^ ^49^, ^50^ ^]^

### 2.6 RegGAIN Identifies Stage‐Specific Driver TF Modules Along hESC Differentiation Trajectory

Our previous analyses focused on differential GRNs derived from distinct conditions (disease versus healthy). To further explore RegGAIN's ability to identify driver TFs in a continuous biological context, we applied RegGAIN to a time‐series scRNA‐seq dataset of hESC differentiation. This dataset includes six time points (0, 12, 24, 36, 72, and 96 h), capturing the dynamic transition from the pluripotent state (0 h), through early differentiation (12–36 h) to lineage‐primed states (72–96 h). GRN was inferred using the entire dataset to obtain a global view of regulatory interactions across all developmental stages. Following GRN inference, we computed AUCell‐based regulon activity scores across individual cells to characterize the temporal regulatory dynamics (Experimental Section).^[^ ^51^ ^]^

**Figure 6:**
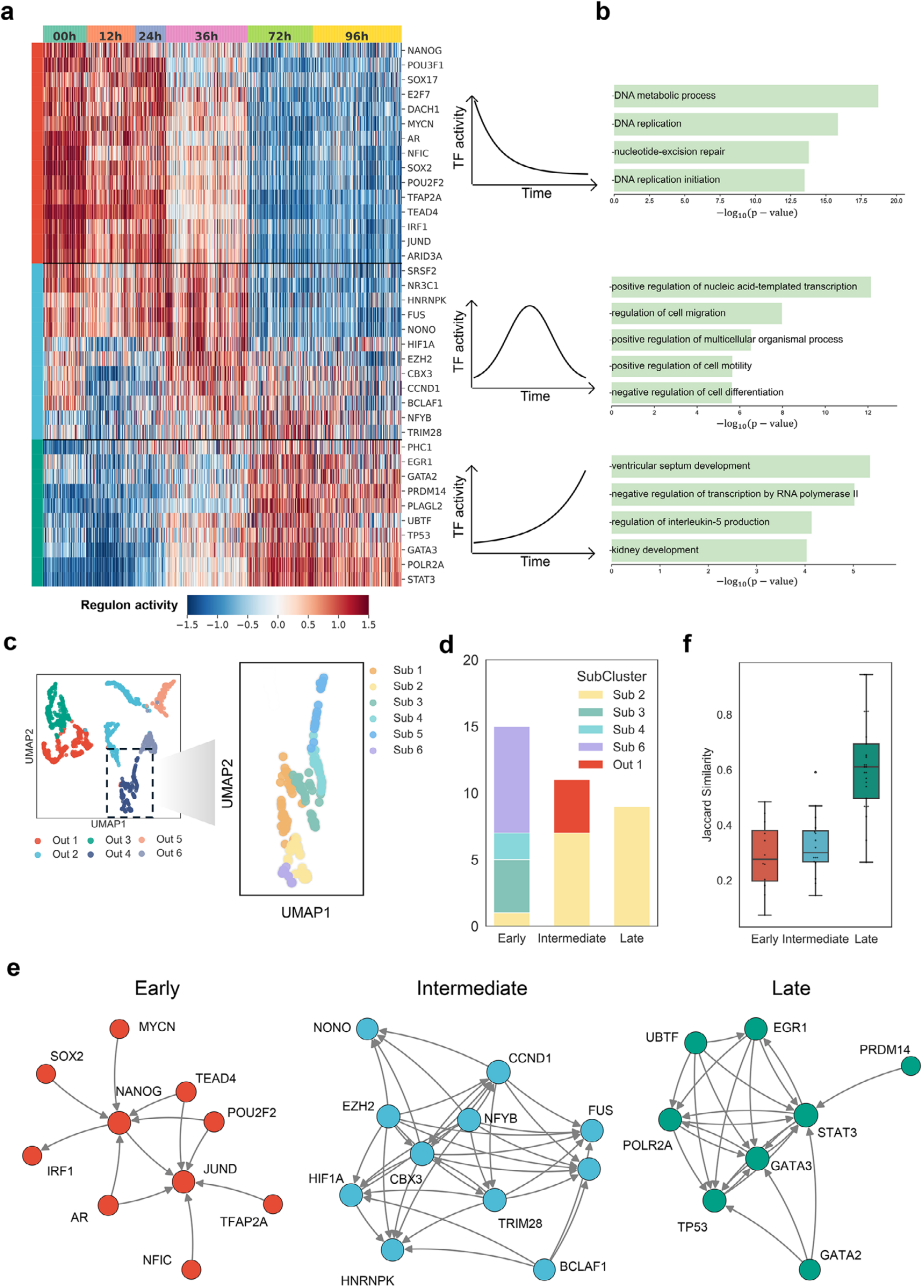
RegGAIN identifies stage‐specific driver TF modules along hESC differentiation trajectory. a) Heatmap of TF regulon activity across individual cells from six time points in the hESC dataset. TFs are grouped into three clusters with distinct temporal activity patterns, schematically represented on the right. b) GO enrichment barplots for the three gene modules, each corresponding to a distinct temporal activity pattern. c) Leiden clustering on all gene out‐embeddings identifies distinct gene groups (left, Out 1–6), visualized in a UMAP projection. A subsequent, finer‐grained clustering was performed on the highlighted population (Out‐4), revealing several sub‐clusters (right, Sub 1–6). d) The barplot shows the distribution of the three TF modules across subclusters. e) Visualization of regulatory sub‐networks among TFs belonging to the three modules. f) The boxplot shows the distribution of Jaccard similarity values for pairwise TF interactions across the three developmental stages.

We focused on TFs showing pronounced variation in regulon activity along the differentiation trajectory and identified three distinct modules, each peaking at early, intermediate, or late stages, respectively (**Figure** **6**a). Gene Ontology (GO) enrichment analysis revealed stage‐specific functional associations for each module: the early module was enriched in DNA replication initiation and nucleotide‐excision repair, reflecting a highly proliferative state;^[^ ^52^ ^]^ the intermediate module was associated with cell migration and organismal development; and the late module was enriched in kidney and heart development, indicating advanced lineage commitment (Figure 6b).^[^ ^53^ ^]^

To further investigate the regulatory structure of these TF modules, we analyzed their distribution within gene clusters derived from the out‐embeddings. A dominant cluster (out‐embedding cluster 4) containing the majority of TFs was subclustered to reveal finer regulatory patterns (Figure 6c). The results showed that TFs from the early module were dispersed across multiple subclusters, suggesting broad and diverse regulatory roles. In contrast, TFs from the intermediate and late modules were more confined, indicating convergent regulation of lineage‐specific programs (Figure 6d). These patterns align with the expected transition from general to specialized regulation during development and differentiation.^[^ ^54^ ^]^

Network‐level analysis also supported these observations, showing increased regulatory convergence and tighter TF subnetworks from early to late stages (Figure 6e,f, average pairwise Jaccard index = 0.29, 0.33, and 0.61, respectively). Late‐stage TFs, in particular, formed dense regulatory circuits with coherent motifs, such as the feed‐forward loop involving *GATA2*, *GATA3*, and *STAT3* (Figure 6e). Consistently, late module TFs like *GATA2* and *GATA3* shared many predicted targets (Jaccard index = 0.48), reflecting their similar binding preferences and cooperative functions (Figure S14, Supporting Information). Together, these findings illustrate a regulatory shift from broad, multipotent control toward coordinated lineage‐specific programs.

## 3 Discussion

Inferring reliable GRNs from scRNA‐seq data remains a fundamental challenge due to pervasive data sparsity, technical noise, and the incompleteness of prior regulatory annotations. Co‐expression‐based approaches are easily confounded by stochastic dropout events, and prior networks are often generic and lack cell‐type specificity. Moreover, traditional graph neural networks are limited in their ability to capture the directionality of transcriptional regulation. To address these issues, we developed RegGAIN, a framework that integrates prior‐guided structural constraints with a self‐supervised contrastive learning paradigm to learn robust and biologically meaningful gene representations.

To address these issues, we present RegGAIN, a self‐supervised graph contrastive learning framework for GRN inference from scRNA‐seq data. Comprehensive benchmarking demonstrated that RegGAIN achieves accurate and robust GRN reconstruction, consistently outperforming existing methods. Its application to complex biological systems, such as identifying dysregulated TFs in multiple myeloma and mapping dynamic regulons during hESC differentiation, underscores its ability to uncover condition‐specific and temporally resolved regulatory programs. Notably, the model's capacity to learn directional relationships also enabled it to uncover biologically meaningful and well‐established regulatory feedback architectures within the inferred GRNs, including core circuits regulate pluripotency maintenance and cell cycle control in hESC (Figure S15, Supporting Information). Unlike traditional supervised paradigms that treat prior knowledge as fixed ground‐truth labels, RegGAIN adopts a fundamentally different philosophy in how prior information is utilized. In a supervised setting, the model directly fits the prior edges as binary labels, which makes it highly sensitive to noise and context mismatches in existing annotations. This can force the model to learn non‐cell‐type‐specific or even incorrect associations. In contrast, RegGAIN adopts a more flexible use of prior knowledge, employing it as a guiding heuristic to construct positive and negative pairs for contrastive learning. The goal is not to replicate prior edges exactly, but to learn meaningful regulatory representations from the data itself. This design encourages the model to capture latent, context‐dependent regulatory patterns, enhances robustness to imperfect prior networks, and promotes generalization across diverse cellular contexts.

A central advance of RegGAIN is its dual‐encoder architecture, which explicitly models the distinct roles of genes as both regulators and targets. This design captures the inherent directionality of GRNs by learning separate embeddings representing regulator‐ and target‐driven patterns. Our analyses confirmed that these dual‐role representations effectively partition regulatory rationale: out‐embeddings clustered TFs with similar downstream targets, while in‐embeddings grouped genes governed by common upstream regulators. This architectural choice directly addresses a critical limitation of conventional GNNs, which often fail to distinguish the asymmetric nature of gene regulation, thereby enabling more precise reconstruction of directed interactions.

The second core innovation lies in RegGAIN's GRN‐aware graph perturbation strategy. Graph contrastive learning has recently emerged as a powerful strategy for representation learning in biological data. Several spatial transcriptomics methods have successfully applied this framework to improve representation learning and downstream analyses.^[^ ^55^, ^56^, ^57^, ^58^ ^]^ Despite their strong performance in spatial transcriptomics, the graph structures they rely on differ fundamentally from gene regulatory networks. Spatial transcriptomics data, such as 10X Visium, form regularly arranged spots whose KNN‐based adjacency graphs show homogeneous degree distributions. Standard contrastive learning often relies on uniform and random augmentations, which can inadvertently disrupt the non‐uniform topology of biological networks. Recognizing that GRNs follow a power‐law distribution, RegGAIN employs a targeted perturbation scheme that differentially corrupts high‐centrality hub nodes and low‐degree nodes. This generates more informative and challenging augmented views, compelling the model to learn representations robust to both the removal of critical regulators and random noise.

Despite its strong performance, RegGAIN still has several limitations. First, its computational runtime can be substantial for very large datasets, reflecting the complexity of its graph‐based deep learning architecture (Figure S16, Supporting Information). This may pose a challenge for users in applications to large‐scale datasets. Second, the current implementation infers one GRN per cell population, requiring separate runs to analyze multiple cell types or conditions. While this supports differential network analysis, it does not inherently capture continuous regulatory transitions between dynamic cell states. Moreover, the current model primarily captures positive regulatory potentials, as edge scores are derived from the similarity between gene embeddings. Distinguishing between activation and repression remains a fundamental challenge in GRN inference based on static transcriptomic data. Future extensions could address this limitation by integrating signed regulatory information from curated databases or by incorporating time‐series and perturbation‐based datasets to enable the explicit learning of regulatory directions and signs.

Besides, several directions could further advance the inference performance of RegGAIN. Integrating additional modalities such as scATAC‐seq or CUT&Tag would provide valuable chromatin accessibility and TF motif information, enabling the construction of high‐confidence, cell‐state‐specific prior networks and thereby enhancing the biological accuracy of inferred GRNs. Furthermore, extending RegGAIN to spatial transcriptomics would facilitate the inference of spatially resolved regulatory networks, offering mechanistic insights into how cell–cell communication and tissue architecture shape transcriptional regulation in development and disease. Another promising direction is extending RegGAIN to perturbation‐based datasets (e.g., CRISPR screens), which could provide causal constraints and further improve the interpretability and biological validity of the inferred networks.

## 4 Experimental Section

### Data Preprocessing

RegGAIN takes as input a single‐cell transcriptomic profile of a cell type or population, along with a species‐specific prior gene interaction network (e.g., human or mouse). Gene expression values were normalized by total counts per gene and log‐transformed to reduce scale differences and improve comparability.^[^ ^59^ ^]^ As the prior network, NicheNet was used,^[^ ^13^ ^]^ a large‐scale knowledge base built by comprehensively integrating numerous biological gene databases, which incorporates information on intracellular signaling, gene regulation, and ligand‐receptor interactions. To focus on intracellular regulation, it was excluded ligand–receptor pairs and extracted a subnetwork containing only genes present in the expression matrix. Genes expressed but absent from the prior network were treated as isolated nodes.

### Details of RegGAIN

In RegGAIN, GRN inference was performed using a graph neural network within a self‐supervised contrastive learning framework.^[^ ^60^ ^]^ This framework first generates two perturbed graph views of the prior network by applying structural and feature corruption. It then encourages embeddings of the same gene across these views to remain consistent while pushing apart embeddings of different genes. To characterize regulatory directionality and account for the distinct roles of genes in regulation, it was implemented separate encoders to learn dual‐role representations for each gene, explicitly capturing its distinct regulator‐driven and target‐driven patterns. Additionally, a high‐order graph convolutional layer is incorporated to model long‐range dependencies within the regulatory network.^[^ ^61^ ^]^ The resulting model produces directed GRNs, where edges denote predicted TF–target regulatory strengths inferred from the learned embeddings.

### Graph Views Generation

Generating diverse graph views was essential for contrastive learning, as it allows the model to maximize information content, obtain comprehensive and robust gene representations, and gain deeper insights into the network topology. In RegGAIN, it was perturb the original prior network G=(V,E)  at both structural and feature levels to create distinct graph views. These perturbations enable the model to learn robust and informative node embeddings by capturing regulatory signals across varied contexts, ultimately enhancing generalization and regulatory inference.

Let $X\in R^{N\times d}$ denote the input feature matrix, where *N* is the number of genes and *d* is the number of cells, and let $A\in R^{N\times N}$ represent the adjacency matrix derived from the prior network. To generate perturbed View 1 $\left({\tilde{X}}_{1},{\tilde{A}}_{1}\right)$, we randomly remove a subset of edges from G with probability *α*_1_, and mask a fraction of the node features to zero with probability *β*_1_.

However, a network's robustness to the above random perturbations is not uniform. It is intrinsically shaped by its underlying topological architecture.^[^ ^62^ ^]^ Biomolecular networks like GRNs often exhibit power‐law degree distributions, characterized by a small number of hub nodes and a large number of low‐degree nodes.^[^ ^63^ ^]^ To ensure meaningful perturbation of such networks, RegGAIN incorporates topological awareness by identifying key substructures within the prior network.^[^ ^64^ ^]^ Specifically, it was computed node centrality scores and select the top‐ranking nodes along with their interactions to form a subgraph $G_{s}=\left(V_{s},E_{s}\right)$, where $V_{s}$ contains the high‐centrality nodes and $E_{s}$ denotes the edges among them. The remaining nodes and their associated edges define a subgraph $G_{r}=\left(V_{r},E_{r}\right)$, representing the less‐central portion of the original graph. Therefore, to achieve more effective perturbation, we design View 2 $\left({\tilde{X}}_{2},{\tilde{A}}_{2}\right)$ by leveraging the power‐law distribution of the network. Structure corruption is applied in a topology‐aware manner: edges in $E_{s}$ are removed with probability *α*_2_, while edges in $E_{r}$ are removed with a different probability *α*_3_. In parallel, node features in $V_{s}$ and $V_{r}$ are independently masked with probabilities *β*_2_ and *β*_3_, respectively.

### Dual‐Role Gene Representation

In conventional GNNs, information propagation was typically one‐directional, with each node aggregating information solely from its downstream neighbors. This limits the model's ability to capture the multifaceted roles of genes, particularly those that act as both regulators and targets. To address this limitation, we model each gene as both a source and a target node, enabling the learning of dual‐role gene representations. Specifically, we implement two separate encoders. The encoder $f_{\mathrm{out}}$ (“out” for outgoing edges) learns the regulator‐driven pattern of a gene by aggregating information from its downstream target genes, while $f_{\mathrm{in}}$ (“in” for incoming edges) learns the target‐driven pattern by aggregating information from its upstream regulators:

(1) $$h^{out}=f_{out}\;\left(X,A\right)$$

(2) $$h^{in}=f_{in}\;\left(X,A^{T}\right)$$

By combining dual‐role gene representation with graph views, RegGAIN generates four distinct embeddings during training—namely, $h^{(1),\mathrm{out}}$, $h^{(1),\mathrm{in}}$, $h^{(2),\mathrm{out}}$, and $h^{(2),\mathrm{in}}$, corresponding to the regulator/target roles under View 1 and View 2, respectively:

(3) $$h^{\left(1\right),\mathrm{out}}=f_{\mathrm{out}}\;\left({\tilde{X}}_{1},\;{\tilde{A}}_{1}\right)$$

(4) $$h^{\left(2\right),\mathrm{out}}=f_{\mathrm{out}}\;\left({\tilde{X}}_{2},\;{\tilde{A}}_{2}\right)$$

(5) $$h^{\left(1\right),\mathrm{in}}=f_{\mathrm{in}}\;\left({\tilde{X}}_{1},\;{\tilde{A}}_{1}^{T}\right)$$

(6) $$h^{\left(2\right),\mathrm{in}}=f_{\mathrm{in}}\;\left({\tilde{X}}_{2},\;{\tilde{A}}_{2}^{T}\right)$$

### High‐Order Graph Convolutional Layer

In GRNs, genes frequently participate in both direct and indirect regulatory interactions, with the latter often occurring through multi‐step pathways involving high‐order neighbors. To effectively capture these complex dependencies, we employ a specialized convolutional layer that aggregates and mixes feature representations across multiple neighborhood orders. The standard GCN layer was defined as:

(7) $$h^{\left(l\right)}=\sigma \left({\hat{A}}_{i}h^{\left(l-1\right)}W^{\left(l-1\right)}\right)$$

(8) $${\hat{A}}_{i}=D_{i}^{-\;\frac{1}{2}}\;\left({\tilde{A}}_{i}+I\right)D_{i}^{-\;\frac{1}{2}},\;i=\left\{1,2\right\}.$$

Here, $h^{\left(l\right)}$ denotes the latent representation of all genes at layer *l*. *σ* is the non‐linear activation function, and $W^{\left(l-1\right)}$ is a trainable weight matrix. ${\hat{A}}_{i}$ is the normalized adjacency matrix for View *i*, where $D_{i}$ is the degree matrix of the perturbed adjacency matrix ${\tilde{A}}_{i}$ and I ***I*** represents the identity matrix introducing self‐loops. In RegGAIN, we extend this layer to incorporate high‐order neighborhood information. Specifically, the convolutional layer was reformulated as:

(9) $$h^{\left(l\right)}={∥}_{j\in S}\;\sigma \left({\hat{A}}_{i}^{j}h^{\left(l-1\right)}W_{j}^{\left(l-1\right)}\right)$$

where S is a set of integers denoting the neighborhood orders to be aggregated (e.g., S={0,1,…,n}), and *n* represents the maximum neighborhood order considered. When S={0}, only the node's own features are used. When S={1}, the formulation reduces to the conventional GCN. When S={0,1,…,n}, RegGAIN integrates multi‐hop neighborhood information, with each order contributing uniquely through its weight matrix $W_{j}^{\left(l-1\right)}$. ${\hat{A}}_{i}^{j}$ represents the *j*‐th power of the normalized adjacency matrix $\;{\hat{A}}_{i}$. The symbol ∥|| denotes column‐wise concatenation across different orders.

### Contrastive Loss Function

In RegGAIN, a contrastive objective function is employed to maximize the agreement between node representations across different graph views. For each gene, four node embeddings are generated corresponding to the regulator/target roles under View 1 and View 2. Within this framework, the embedding of a gene under one view (e.g., $h_{i}^{(1),out}$) is designated as the anchor, while the embeddings of the same gene under two views ($h_{i}^{(1),out},h_{i}^{(2),out}$) serve as positive samples. To enforce representation consistency, embeddings of different genes—regardless of whether they come from the same or different views—were treated as negative samples (e.g., $h_{i}^{(1),out}$ versus $h_{k}^{(1),out}$ and $h_{k}^{(2),out}$, where i≠k).

The pairwise contrastive loss for each positive pair is defined as:

(10) $$\begin{array}{cc} & L^{out}\left(h_{i}^{\left(1\right),out},h_{i}^{\left(2\right),out}\right)=\\ & \;-\log\frac{S\left(h_{i}^{\left(1\right),out},h_{i}^{\left(2\right),out}\right)}{S\left(h_{i}^{\left(1\right),out},h_{i}^{\left(2\right),out}\right)+\sum_{k\;=1}^{N}1_{k\neq i}\left[S\left(h_{i}^{\left(1\right),out},h_{k}^{\left(1\right),out}\right)+S\left(h_{i}^{\left(1\right),out},h_{k}^{\left(2\right),out}\right)\right]} \end{array}$$

(11) $$\begin{array}{cc} & L^{in}\left(h_{i}^{\left(1\right),in},h_{i}^{\left(2\right),in}\right)=\\ & \;-\log\frac{S\left(h_{i}^{\left(1\right),in},h_{i}^{\left(2\right),in}\right)}{S\left(h_{i}^{\left(1\right),in},h_{i}^{\left(2\right),in}\right)+\sum_{k\;=1}^{N}1_{k\neq i}\left[S\left(h_{i}^{\left(1\right),in},h_{k}^{\left(1\right),in}\right)+S\left(h_{i}^{\left(1\right),in},h_{k}^{\left(2\right),in}\right)\right]} \end{array}$$

Here, S(·,·)=exp(cos(g(·),g(·))/τ),  where g(·) is a two‐layer non‐linear projection, and τ is a temperature parameter. Cosine similarity is used to quantify the similarity between two embeddings. The indicator $\;1_{k\neq i}$ ensures that only negative pairs (i.e., k≠i) are included in the denominator. As the two views are treated symmetrically, the contrastive loss is computed in both directions. The total loss is obtained by averaging the loss over all genes:

(12) $$L\;\left(h_{i}^{\left(1\right)},h_{i}^{\left(2\right)}\right)=L^{out}\;\left(h_{i}^{\left(1\right),out},h_{i}^{\left(2\right),out}\right)+L^{in}\left(h_{i}^{\left(1\right),in},h_{i}^{\left(2\right),in}\right)$$

(13) $$L_{\mathrm{Total}}=\frac{1}{2N}\;\sum_{i\;=1}^{N}\left[L\left(h_{i}^{\left(1\right)},h_{i}^{\left(2\right)}\right)+L\left(h_{i}^{\left(2\right)},h_{i}^{\left(1\right)}\right)\right]$$

### Gene Regulatory Network Construction

Upon completion of neural network training, the final gene embeddings are obtained as: $h^{\mathrm{out}}=f_{\mathrm{out}}\left(X,A\right),h^{\mathrm{in}}=f_{\mathrm{in}}\left(X,A^{T}\right)$. To estimate the potential regulatory strength between each gene pair, we first normalized these embeddings to obtain ${\hat{h}}^{\mathrm{out}}$ and ${\hat{h}}^{\mathrm{in}}.$ After this, we construct a regulatory score matrix $M\in R^{N\times N}$ as:

(14) $$M=P\circ {\hat{h}}^{\mathrm{out}}\cdot {\left({\hat{h}}^{\mathrm{in}}\right)}^{T}$$

where each entry in M reflects the interaction strength from a putative regulator to its target. $P\in R^{N\times N}$ is a weighting matrix defined by the prior network structure. Each element $p_{\mathrm{ij}}$ in P is set to a constant γ > 1 if the edge (*i*, *j*) exists in the prior network, and to 1 otherwise. ∘ denotes the Hadamard (element‐wise) product. This formulation leverages learned embeddings while encouraging consistency with known prior interactions.

To mitigate the impact of randomness, the inference process was repeated ten times, and the regulatory score matrices were averaged across runs to obtain robust estimates. Ultimately, the top *H* highest‐scoring edges between TFs and their putative target genes are selected to construct the inferred GRN (default 10*number of genes).

### RegGAIN Implementation

In RegGAIN, the encoder was a three‐layer GNN incorporating high‐order convolutions. At each layer, we set S={0,1,2} to aggregate information from 0‐hop, 1‐hop, and 2‐hop neighbors. In the first layer, the output dimensions were set to [80, 80, 10], respectively. The subsequent two hidden layers used dimensions of [40, 40, 5] and [16, 16, 2], respectively. This configuration results in a final gene embedding of size 34 (16+16+2). A Tanh activation function was applied after each GNN layer. The resulting embeddings are then passed through a two‐layer projection head with a hidden dimension of 64 and an ELU activation function, producing outputs of the same dimension as the input for contrastive loss calculation.

The model trained for 500 epochs by default and was optimized using the Adam optimizer with a learning rate of 0.001. The temperature parameter τ for the contrastive loss function was set to 3. For data augmentation, feature drop rates were set to 0.5 on high‐degree nodes and 0.2 for low‐degree nodes (*β*_1_ =  0.2,   *β*_2_ =  0.5,   *β*_3_ =  0.2). Structure corruption was applied with edge drop rates of 0.6 for high‐centrality edges and 0.3 for other edges (*α*_1_ =  0.3,   *α*_2_ =  0.6,   *α*_3_ =  0.3). To distinguish node types, we identified hub nodes as those in the top 15% of the degree centrality distribution within the prior network, and the weighting parameter γ was set to 10 to emphasize prior interactions in benchmark (Figures S17 and S18, Supporting Information). For broader exploration of potential regulatory of potential regulatory relationships or differential regulation, γ can be reduced (e.g., γ  =  1).

### Evaluation Metrics

To quantitatively assess the accuracy of inferred GRNs, two widely‐used evaluation metrics was employed: Area Under the Precision‐Recall Curve Ratio (AUPRC ratio) and Early Precision Ratio (EPR). The AUPRC ratio reflects the overall trade‐off between precision and recall under class imbalance, normalized by the expected performance of a random predictor. EPR measures precision among top‐ranked predictions relative to a random baseline, emphasizing early retrieval performance. Detailed formulas for these evaluation metrics were provided in Supporting Information.

### Robustness Experiments Design

In this study, we designed two perturbation experiments to evaluate the robustness of RegGAIN against common data imperfections. First, to assess the model's tolerance to incomplete prior knowledge, we randomly removed 10% to 50% of edges from the prior network before inference. Second, to test its performance under reduced cellular resolution, we conducted a parallel experiment by randomly removing a fraction of cells (ranging from 10% to 50%) from the input expression matrix. Third, to evaluate its resilience to false‐positive edges, we corrupted the prior network by replacing 5% to 70% of true edges with randomly selected false edges. This noise was introduced using two strategies: 1) a simple random edge swapping and 2) a more rigorous degree‐preserving swapping to maintain the network's structural properties. To account for the variability introduced by these perturbations, each condition at every removal level was independently repeated 10 times. The final AUPRC ratio and EPR scores represent the average performance across these replicates.

### Clustering Analysis

It was clustered genes based on their regulatory embeddings (i.e., $h^{\mathrm{out}}$ and $h^{\mathrm{in}}$) learned from RegGAIN. Embeddings were standardized and visualized via the uniform manifold approximation and projection (UMAP). Clustering was performed using K‐means (num_clusters = 6) and the Leiden algorithm with resolutions of 0.2 and 1.0. All clustering analyses were conducted with a fixed random seed to ensure reproducibility. To validate the robustness of our conclusions, we performed a sensitivity analysis, which demonstrated that the observed functional separation of the embeddings remains stable across different numbers of clusters (Figure S7, Supporting Information).

To assess the consistency of clustering results in Figure 3d, it was calculated pairwise Jaccard similarities between TF pairs. Specifically, predicted target genes for each TF were first derived from the reconstructed GRN, and the Jaccard indices were calculated for each TF pair based on their target genes. The top 500 similar TF pairs were then selected and the proportions of pairs assigned to the same cluster under out‐ and in‐embedding clusters were measured separately. It was then measured the cumulative proportion of these pairs that were assigned to the same cluster in out‐embedding versus in‐embedding spaces. An analogous analysis was performed for target genes by computing Jaccard similarities based on their upstream regulators in Figure 3e. To ensure that our findings were not specific to this metric, we confirmed that similar results were obtained using alternative metrics, including Cosine, Dice, and Kulczynski similarities (Figure S8, Supporting Information).

### TF Motif Enrichment Analysis

To evaluate the biological relevance of predicted TF–target interactions, we conducted motif enrichment analysis using FIMO,^[^ ^65^ ^]^ applying a stringent significance threshold of *p* < 1e–4. We focused on high‐confidence regulatory interactions by selecting the top 5% of TF–target pairs ranked by predicted regulatory score. For each target gene, candidate regulatory regions were defined as ±200 kb around the transcription start site, within which motif occurrences were scanned. A sensitivity analysis demonstrated that our results remain robust across a broad range of window sizes (Figure S19, Supporting Information). A curated database of vertebrate TF motifs (e.g., JASPAR^[^ ^66^ ^]^) was used as input for FIMO, and TFs were linked to their corresponding DNA‐binding motifs based on annotations provided in the database.

Specifically, for each target gene *g*, we calculated a “TF hit ratio” to quantify motif‐level support for its predicted regulators. This ratio is defined as the fraction of predicted TFs for which FIMO detects at least one corresponding binding motif within the gene's candidate regulatory region. Formally, the hit ratio for gene *g* is given by:

(15) $$\mathrm{Hit}\;\mathrm{Rati}o_{g}=\frac{\mathrm{Number}\;\mathrm{of}\;\mathrm{Predicted}\;\mathrm{TFs}\;\mathrm{for}\;\mathrm{gene}\;g\;\mathrm{with}\;a\;\mathrm{Motif}\;\mathrm{Hit}}{\mathrm{Total}\;\mathrm{Number}\;\mathrm{of}\;\mathrm{Predicted}\;\mathrm{TFs}\;\mathrm{for}\;\mathrm{gene}\;g}\;$$

In addition to the mean hit ratio, we also assessed the proportion of target genes with non‐zero hit ratios—i.e., those for which at least one predicted TF–target interaction is supported by a motif hit (Hit Ratio > 0). This metric reflects the overall coverage of regulatory predictions that are supported by motif‐level evidence.

For comparison, we applied the same evaluation procedure to targets selected by two alternative random methods: 1) Random Selection, where TFs were randomly sampled for each target gene; and 2) NicheNet, where TFs were randomly chosen from the pool of candidate TFs associated with each target gene in the prior network provided by NicheNet. In both baselines, the number of TFs per target gene was matched to that predicted by RegGAIN to ensure a fair comparison.

### Ranking TFs

To investigate gene regulatory network rewiring in MM, we inferred TF–target regulatory scores for each sample. We then applied the Wilcoxon rank‐sum test to identify interactions that show significant differences between MM and normal samples (Figure 5a,b). To prioritize key TFs, we ranked all TFs based on their differential out‐degree, defined as the number of significantly upregulated regulatory edges minus the number of significantly downregulated edges for each TF (Figure 5c).

### MM Signature Score Calculation

A pseudo‐regulon was built based on the top 10 significantly upregulated and downregulated target genes identified by RegGAIN. These genes served as the positive and negative target genes of the constructed pseudo‐regulon, respectively. The single‐sample extension of MARINa (function name: viper) in the viper R package (version 1.42.0) was employed to infer the activity of the constructed pseudo‐regulon, which was used as the signature score for each sample.^[^ ^67^ ^]^

### Survival Analysis

To evaluate the clinical relevance of top‐rewired TFs identified by RegGAIN, it was performed survival analysis using bulk RNA‐seq and clinical data from the MMRF CoMMpass cohort (https://research.themmrf.org/). Gene expression was Z‐score normalized across patients, and samples were stratified into high‐ and low‐expression groups based on the cohort mean. Survival curves of these two groups were estimated by the Kaplan–Meier method with statistical significance calculated using the log‐rank test, and hazard ratios were obtained using a univariate Cox proportional hazards model. All analyses were performed using the survival (version 3.8.3) and survminer (version 0.5.0) R packages.

### Pathway Enrichment Analysis

GO enrichment analysis was conducted using the Enrichr function from the gseapy Python package (version 0.10.8) for gene clusters or modules. Statistical significance was assessed using the hypergeometric test, and *p*‐values were adjusted using the false discovery rate (FDR) method. Besides, pre‐ranked GSEA was performed using the gseapy Python package (version 0.10.8) with the Hallmark gene sets from MSigDB (v2020). For each target gene, its differential regulation score by a score defined as the difference between the number of significantly upregulated and downregulated TF–target edges was quantified. This ranked list was then used as input to GSEA, which was run with 1000 permutations and a minimum gene set size of 15.

### Regulon Activity Score Calculation

To quantify the activity of key transcriptional programs in Figure 6a, we first identified “driver TFs” as those with the top 100 highest out‐degree in the predicted GRN. For each driver TF, we calculated the per‐cell activity of its regulon using the aucell function from the pyscenic Python package, followed by Z‐score normalization. TFs were then selected for visualization based on a one‐way analysis of variance (ANOVA) of their regulon activity across developmental time points, retaining those with *p*‐values < 0.05.^[^ ^68^, ^69^ ^]^

### Statistical Analysis

Statistical significance for TF cluster enrichment was determined using Fisher's exact test. GO enrichment was assessed with the hypergeometric test, and the resulting p‐values were adjusted for multiple comparisons using the FDR. Pathway enrichment was evaluated via the pre‐ranked GSEA permutation test. Survival analyses were conducted using the log‐rank test, while the ANOVA was applied to identify TFs with significant temporal variation. All comparisons between two groups, including method performance metrics (e.g., AUPRC ratio and EPR), differential regulatory strength, and TF motif hit ratios, were performed using the two‐sided Wilcoxon rank‐sum test.

## Conflict of Interest

The authors declare no conflict of interest.

## Author Contributions

D.S. and L.Y.W. conceived the idea and supervised the study. Q.G. implemented the algorithm and performed the analyses. Q.G., D.S., and L.Y.W. interpreted the results. J.Y., J.P., F.Y., J.J., R.Z., Z.P.L., and B.L. provided scientific insights on the applications. Q.G. and D.S. wrote the manuscript with feedback from all other authors. All authors read and approved the final manuscript.

## Supporting information



Supporting Information

## Data Availability

Data sharing is not applicable to this article as no new data were created or analyzed in this study.
