# Supplementary material for: Inferring Gene Regulatory Networks From Single‐Cell RNA Sequencing Data by Dual‐Role Graph Contrastive Learning
Source: Adv Sci (Weinh). 2025 Nov 29;13(9):e18277. doi: 10.1002/advs.202518277 (PMC12904006; doi:10.1002/advs.202518277)
Supplement: Supplementary file 1 — Supporting Information [file ADVS-13-e18277-s001.pdf]

Supporting Information

Inferring gene regulatory networks from single-cell RNA sequencing data by dual-role graph contrastive learning

Qiyuan Guan[1], Jiating Yu[2], Jieyi Pan[1], Fan Yuan[3], Jiadong Ji[4, 5], Rusong Zhao[6], Zhi-Ping Liu[7], Bingqiang Liu[1], Ling-Yun Wu[8, 9, *], and Duanchen Sun[1, 5, *]

1 School of Mathematics, Shandong University, Jinan 250100, China;
2 School of Mathematics and Statistics, Nanjing University of Information Science & Technology, Nanjing 210044, China;
3 School of Mathematics and Information Science, Yantai University, Yantai 264005, China;
4 Institute for Financial Studies, Shandong University, Jinan 250100, China;
5 Shandong Key Laboratory of Cancer Digital Medicine, Jinan 250033, China;
6 State Key Laboratory of Reproductive Medicine and Offspring Health, Center for Clinical Reproductive Medicine, the First Affiliated Hospital of Nanjing Medical University, Center for Reproductive Medicine, Institute of Women, Children and Reproductive Health, Shandong University, Jinan 250012, China;
7 Department of Biomedical Engineering, School of Control Science and Engineering, Shandong University, Jinan, Shandong 250061, China;
8 State Key Laboratory of Mathematical Sciences, Academy of Mathematics and Systems Science, Chinese Academy of Sciences, Beijing 100190, China;
9 School of Mathematical Sciences, University of Chinese Academy of Sciences, Beijing 100049, China.

* These authors jointly supervised this work: Ling-Yun Wu, Duanchen Sun. Email: lywu@amss.ac.cn, dcsun@sdu.edu.cn.

1. Supplementary Notes

1.1 Supplementary Note 1

In this section, we describe the datasets utilized throughout this study for model benchmarking, epigenomic validation, and disease-specific analysis.

1.1.1 Benchmark datasets

To systematically evaluate the predictive performance of our proposed model, we adopted the BEELINE[1] evaluation framework and applied it to seven diverse single-cell RNA sequencing (scRNA-seq) datasets from different biological systems. These datasets include human embryonic stem cells (hESC), human mature hepatocytes (hHEP), mouse embryonic stem cells (mESC), mouse dendritic cells (mDC), and three mouse hematopoietic stem cell lineages, namely erythroid (mHSC-E), granulocyte–macrophage (mHSC-GM), and lymphoid (mHSC-L) lineages.

For each experimental dataset, we established three categories of ground-truth gene regulatory networks based on their respective data sources: (1) cell-type-specific Chromatin Immunoprecipitation Sequencing (ChIP-seq) data, (2) non-cell-type-specific ChIP-seq data, and (3) functional interaction networks curated from the STRING database. These resources capture complementary aspects of gene regulation—ChIP-seq reflects direct transcription factor (TF)–DNA binding (either specific or general), while STRING provides broader, often indirect functional associations. Together, they enable a comprehensive evaluation of model performance across both context-specific and general regulatory relationships.

Following the established protocol described in BEELINE, we performed standardized preprocessing on each scRNA-seq dataset, with a specific focus on inferring transcriptional interactions originating from TF. For GRN inference, we implemented a rigorous gene selection procedure by identifying significantly variable genes based on stringent statistical criteria. Specifically, we selected two distinct gene sets comprising 500 and 1,000 most highly variable genes (HVGs), respectively. The selection criterion required that all included TFs demonstrate statistically significant variation, as determined by a Bonferroni-corrected *p*-value threshold of <0.01 for gene expression variance.

1.1.2 Datasets for epigenome validation

To assess the epigenetic plausibility of RegGAIN-inferred regulatory interactions, we utilized the Gene Expression Omnibus (GEO) multi-omics dataset GSE159623, which includes scRNA-seq, single-cell Assay for Transposase-Accessible Chromatin using Sequencing (scATAC-seq), and epigenomic profiles from mESCs. scATAC-seq reads were aligned to the mm10 genome with Tn5 offset correction and peaks called using MACS2. ChIP-seq data for Nanog and Pou5f1 were obtained from ENCODE. We further integrated bigwig files for multiple histone marks (H3K4me3, H3K27ac, H3K4me1, H3K27me3, H3K9me3) to evaluate whether predicted TF–target gene pairs co-localized

with active or repressive chromatin states.

1.1.3 Multiple Myeloma datasets

We analyzed scRNA-seq data from the GEO dataset GSE193531, focusing on two groups: normal bone marrow (NBM) and multiple myeloma (MM). Preprocessing was performed using the Scanpy pipeline. Samples with insufficient cell numbers were removed, resulting in 8 high-quality samples per group. HVGs were selected separately for each group. Given our focus on gene regulatory analysis, we prioritized the inclusion of TFs by applying a higher variability threshold to TFs. In total, 1,523 genes were selected, including 603 TFs. MMRF CoMMpass data used in this study can be obtained from the MMRF Research Gateway [https://research.themmrf.org].

1.2 Supplementary Note 2

In this section, we provide a brief overview of the baseline methods and prior interaction networks used in our benchmarking experiments.

1.2.1 Brief introduction of the baseline methods

GRNBoost2[2]. GRNBoost2 is a widely adopted tree-based method for GRN inference. It models gene expression using gradient boosting machines, treating each gene as a response variable predicted by a set of candidate regulators (typically TFs). GRNBoost2 is entirely data-driven and does not incorporate prior biological knowledge.

DeepSEM[3]. DeepSEM is a deep learning framework based on structural equation modeling. It integrates multi-layer neural networks with sparsity-inducing penalties to infer causal regulatory relationships. DeepSEM captures non-linear dependencies between regulators and targets but does not utilize prior interaction networks.

KEGNI[4]. KEGNI is a knowledge-guided framework for cell-type-specific GRN inference based on scRNA-seq data. It employs a Masked Graph Autoencoder (MAE) to capture gene regulatory relationships from expression profiles and a Knowledge Graph Embedding (KGE) model that integrates prior biological knowledge from KEGG and CellMarker databases. Through multi-task learning optimizing both MAE and KGE objectives, KEGNI enables construction of cell-type-specific GRNs, supports identification of driver genes in regulatory networks, and operates without reliance on paired scRNA-seq/scATAC-seq data.

NetREX[5]. NetREX is a prior-guided method that reconstructs condition-specific GRNs by integrating gene expression data with a fixed, context-agnostic prior network. It introduces latent TF activity variables and solves a constrained optimization problem that selectively rewires the prior network to better match the observed data. The optimization is performed using the PALM algorithm, balancing prior consistency and data fidelity through regularization.

CEFCON[6]. CEFCON is a context-aware GRN inference method that combines prior interaction networks with single-cell transcriptomic data using a graph attention network. It employs contrastive learning to enhance edge specificity across cellular states, and further applies control-theoretic influence scoring to identify key TFs regulating cell fate transitions. CEFCON effectively integrates expression dynamics with network priors.

Random NicheNet[7]. Random NicheNet serves as a negative control. It randomly samples the same number of edges as the inferred network from the NicheNet prior, ignoring gene expression. This method helps evaluate whether GRN models extract meaningful regulatory signals beyond prior structure alone.

1.2.2 Brief introduction of the different prior gene interaction networks

NicheNet[7]. NicheNet is a ligand–target regulatory network originally developed to model intercellular communication. It integrates diverse sources of prior knowledge,

including protein–protein interactions, signaling pathways, and transcriptional regulation, to construct a weighted, directed network linking ligands to their downstream targets. Although NicheNet was not specifically designed for gene regulatory network reconstruction, it provides a valuable resource of regulatory relationships. The data used in this study were downloaded from the NicheNet GitHub repository (https://github.com/saeyslab/nichenetr/tree/master/data).

NicheNet_Directed. NicheNet_Directed is a filtered version of the NicheNet network that retains only the interactions with defined directionality (e.g., from regulator to target).

PathwayCommons[8]. PathwayCommons aggregates curated biological pathway data from resources like Reactome, KEGG, and BioGRID. The extracted regulator–target network contains ~1,000,000 directed edges covering transcriptional regulation and signaling events with literature-based support. For this study, we obtained the network data from https://www.pathwaycommons.org/.

InBiomap[9]. InBioMap is a protein–protein interaction (PPI) database integrating high-confidence experimental and predicted interactions. The network used includes ~600,000 interactions between genes, capturing co-functional and physical associations among proteins. For this study, we obtained the network data via the Omnipath Python package, using the in_biomap_download function.

Harmonizome[10]. Harmonizome compiles functional associations from over 100 datasets, including gene co-expression, perturbation responses, and transcription factor binding. The resulting network comprises approximately 3,000,000 gene–gene associations, providing a broad and heterogeneous prior knowledge base. For this study, we obtained the network data via the Omnipath Python package, using the nichenet_gr_network_harmonizome and nichenet_signaling_network_harmonizome functions.

Omnipath[11]. OmniPath provides a curated set of directed molecular interactions, including post-translational modifications, transcriptional regulation, and signaling pathways. The network used here includes ~80,000 directed interactions, with signed edges indicating activation or inhibition where available. For this study, we obtained the network data via the Omnipath Python package, using the import omnipath interactions functions.

1.3 Supplementary Note 3

In this section, we will introduce two widely-used quantitative metrics—Early Precision Ratio (EPR) and Area Under the Precision-Recall Curve Ratio (AUPRC ratio)—to rigorously assess the performance of our inferred GRNs.

1.3.1 Early Precision Ratio (EPR)

We define early precision as the fraction of true positive interactions among the top-k predicted edges, where k corresponds to the number of edges in the ground-truth regulatory network:

$$EP = \frac{Number\ of\ true\ positives\ among\ top\ k\ predictions}{k}.$$

To normalize early precision and account for the sparsity of the ground-truth network, we compute the EPR, defined as the ratio between the model's early precision and that of a random predictor. The expected early precision of a random predictor corresponds to the edge density of the ground-truth network:

$$EP_{random} = \frac{k}{n(n-1)},$$

where n is the number of nodes (genes).

The EPR is defined as:

$$EPR = \frac{EP_{model}}{EP_{random}}.$$

EPR greater than 1 indicates that the model's predictions outperform random guessing.

1.3.2 Area Under the Precision-Recall Curve ratio (AUPRC ratio)

To assess overall prediction performance while accounting for class imbalance, we compute the AUPRC for each predicted GRN. This metric summarizes the trade-off between precision and recall across all decision thresholds, offering a comprehensive view of model performance.

To normalize the AUPRC and enable fair comparisons across datasets with varying network densities, we define the AUPRC ratio as the ratio between the model's AUPRC and the expected AUPRC of a random predictor. For a random classifier, the expected AUPRC equals the proportion of true regulatory edges among all possible gene pairs, i.e., the edge density of the ground-truth network:

$$AUPRC_{random} = \frac{k}{n(n-1)},$$

where k is the number of true edges in the ground-truth network and n is the number of nodes (genes).

The AUPRC ratio is then defined as:

$$AUPRC\ ratio = \frac{AUPRC_{model}}{AUPRC_{random}}.$$

AUPRC ratio greater than 1 indicates that the model's predictions outperform random guessing.

2. Supplementary Figures

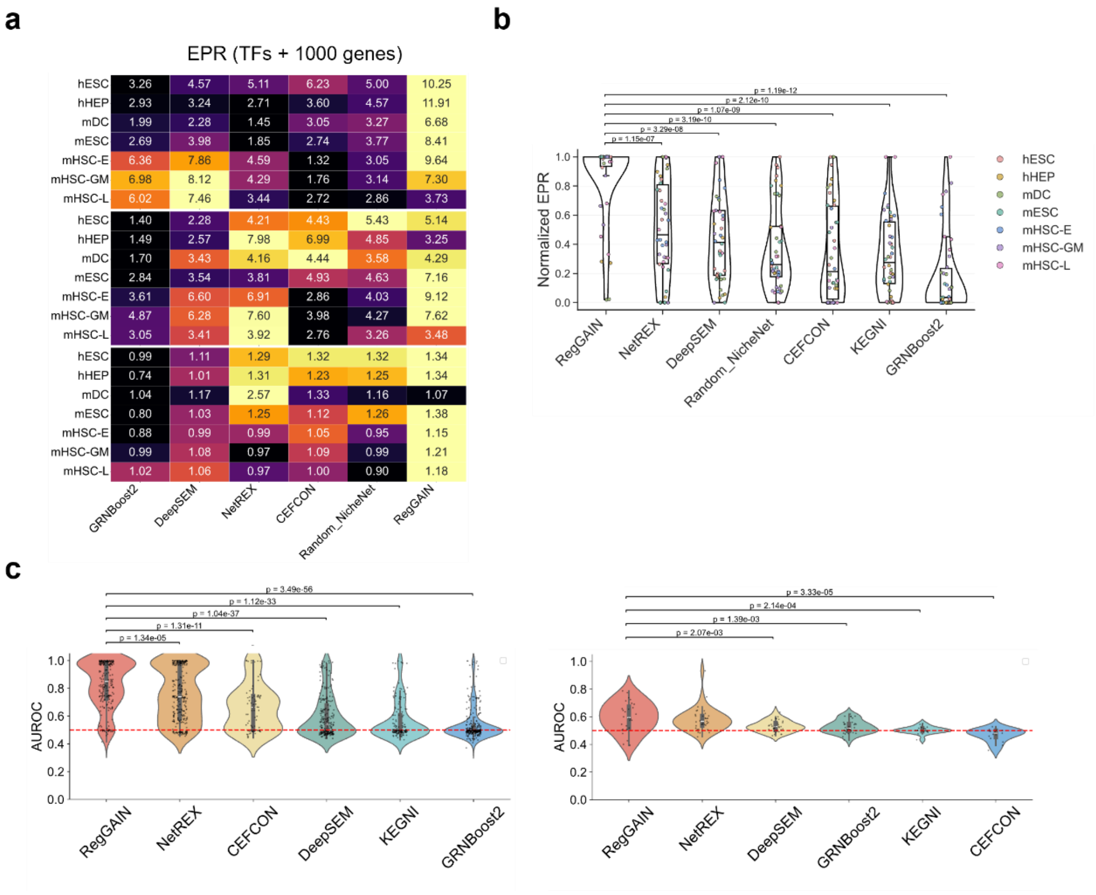

Figure S1. a, Heatmap shows the EPR performance of GRN inference on seven scRNA-seq datasets, evaluated against three distinct ground-truth sources, using the 1,000 most variable genes and all variable TFs. **b**, Violin plots show the distributions of normalized EPR across different ground-truth networks and datasets. Each colored dot represents the performance score on one of the seven datasets. Statistical significance was assessed using the Wilcoxon rank-sum test, comparing the performance of RegGAIN with that of each other method. **c**, Violin plots illustrate the distributions of AUROC scores achieved by each model for predicting the targets of individual transcription factors, evaluated using the Non-Specific (left) and cell-type-specific (right) ground-truth network. Statistical significance was evaluated using the Wilcoxon rank-sum test, comparing the performance of RegGAIN with that of other method.

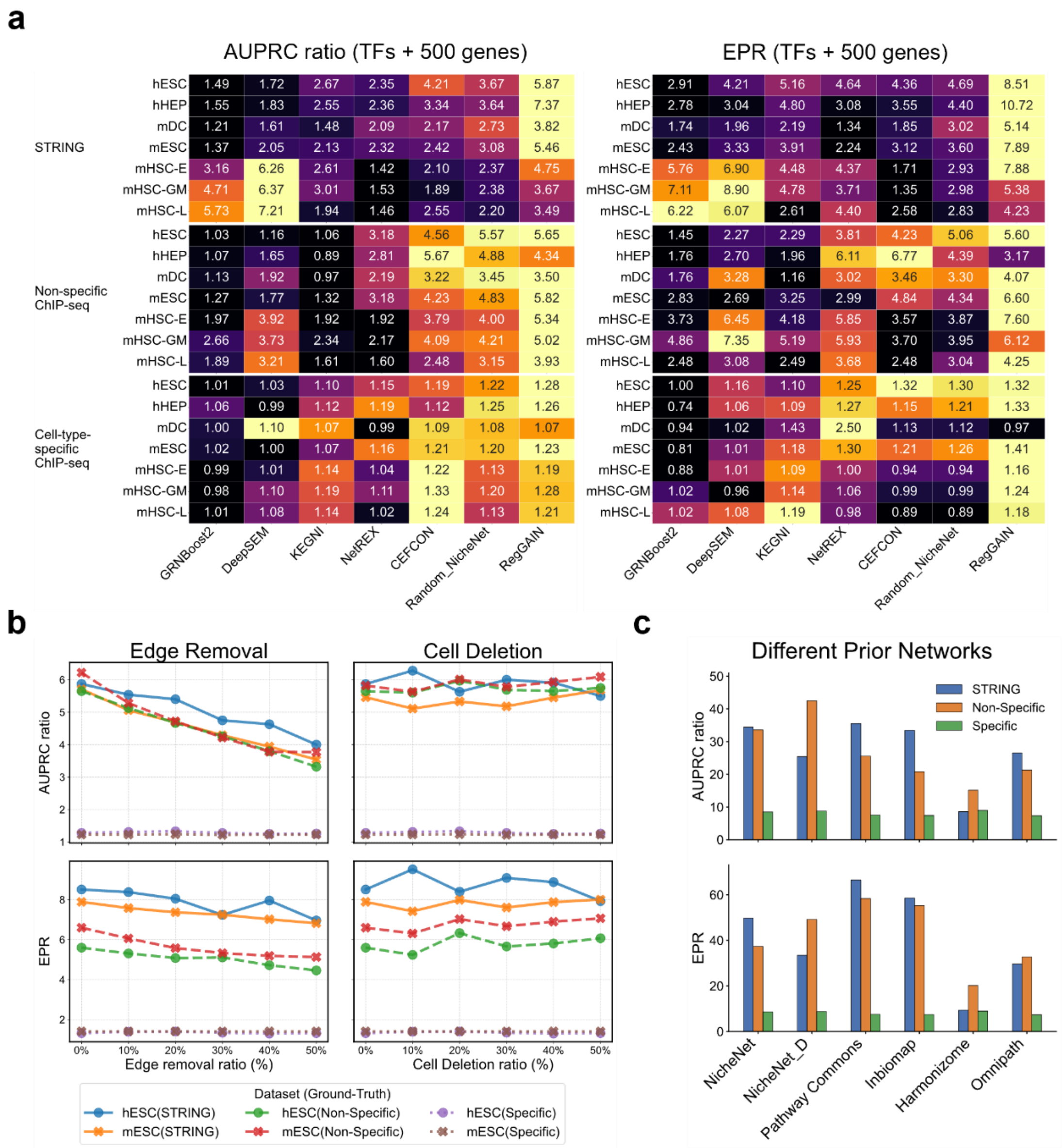

Figure S2. a, Heatmap shows the AUPRC ratio and EPR performance of GRN inference on seven scRNA-seq datasets, evaluated against three distinct ground-truth sources, using the 500 most variable genes and all variable TFs. **b,** Robustness of RegGAIN under perturbations, assessed by progressively removing edges from the prior network (left) or deleting cells from the scRNA-seq data (right) using TF+500 HVGs on hESC and mESC datasets with three ground-truth sources (Top: AUPRC ratio; Bottom: EPR). **c**, Overall performance of RegGAIN (Top: AUPRC ratio; Bottom: EPR) across seven datasets using six different prior networks.

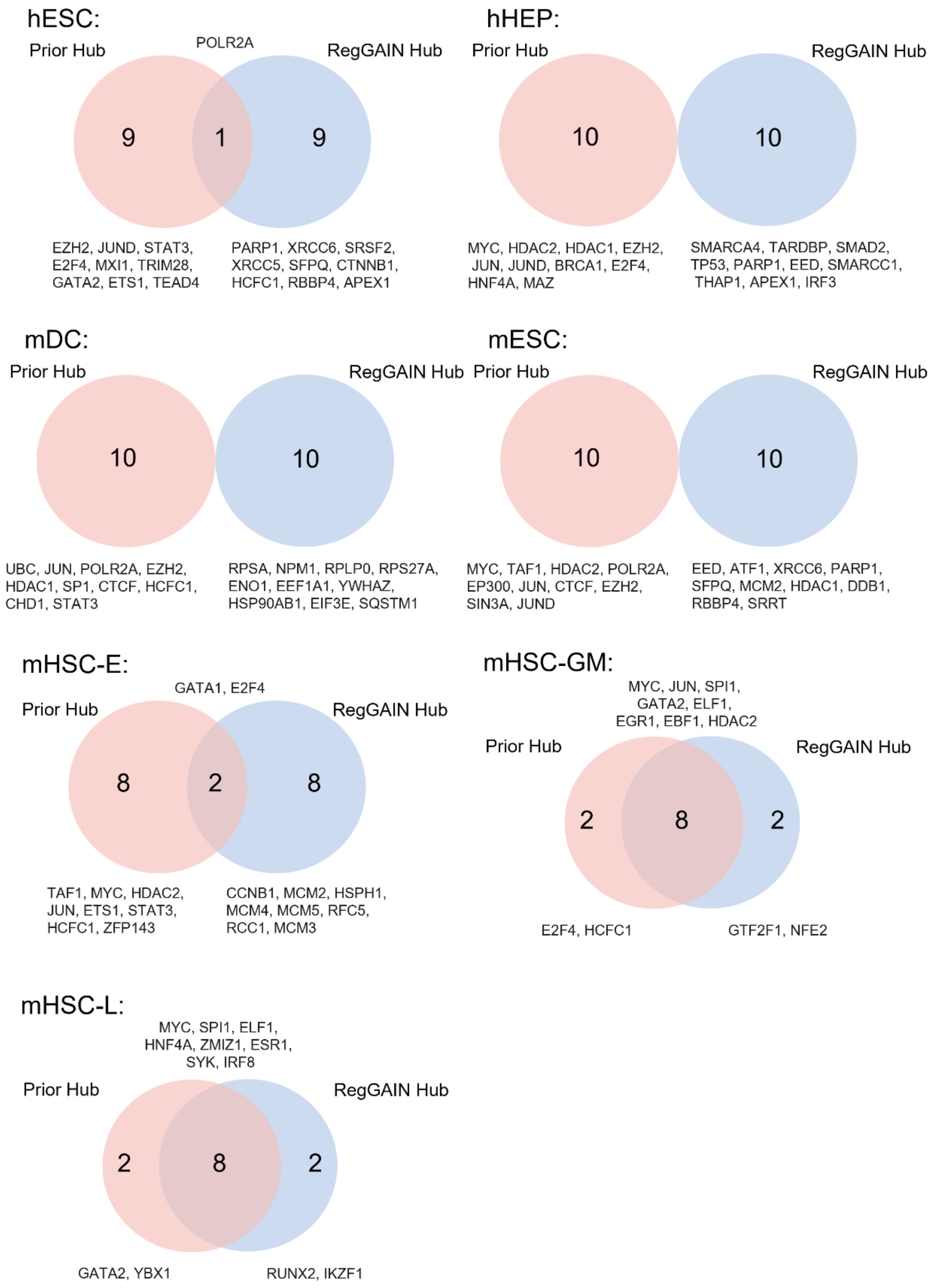

Figure S3. Overlap of hub genes identified by prior networks and RegGAIN-inferred networks across seven datasets. Venn diagrams show the hub genes (top 10 by degree centrality) derived from prior networks (red) and RegGAIN-predicted networks (blue) for each dataset, including hESC, hHEP, mDC, mESC, mHSC-E, mHSC-GM, and mHSC-L. Numbers inside the circles indicate the count of unique and shared hub genes, with gene names listed below each diagram.

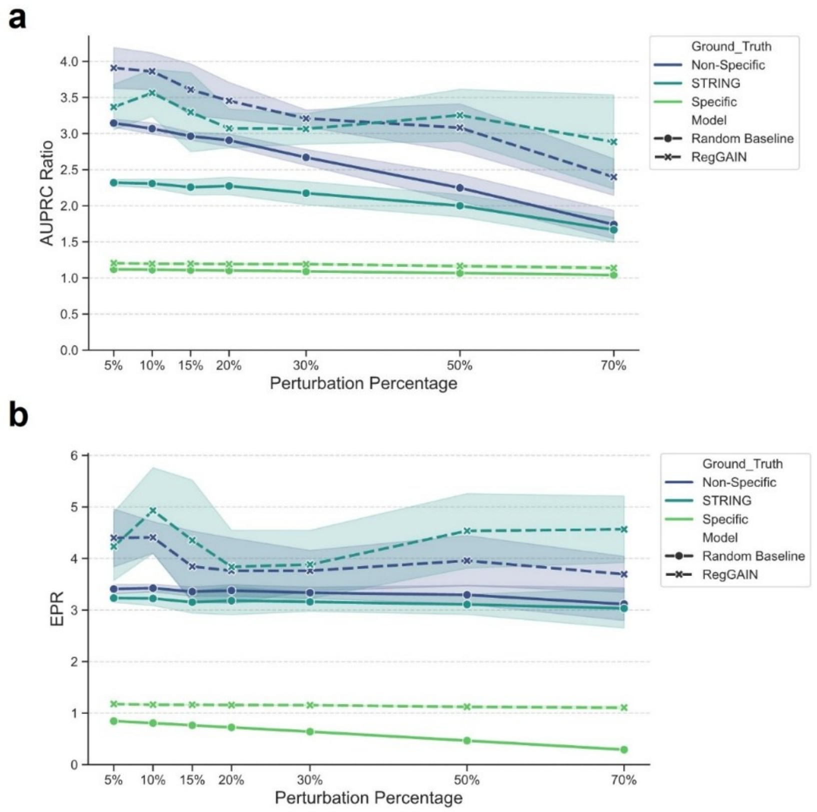

Figure S4. Robustness of RegGAIN against edge replacement perturbations in the prior network. The plots show RegGAIN's performance, measured by (**a**) AUPRC ratio and (**b**) EPR, as an increasing percentage of true edges in the prior network are replaced with random false edges. This perturbation strategy simulates noise by removing a specific fraction of known interactions and introducing an equal number of spurious ones. Dashed lines with 'x' markers represent the mean performance of RegGAIN, compared to a baseline model (solid lines with 'o' markers) that uses only the corrupted prior network for its predictions.

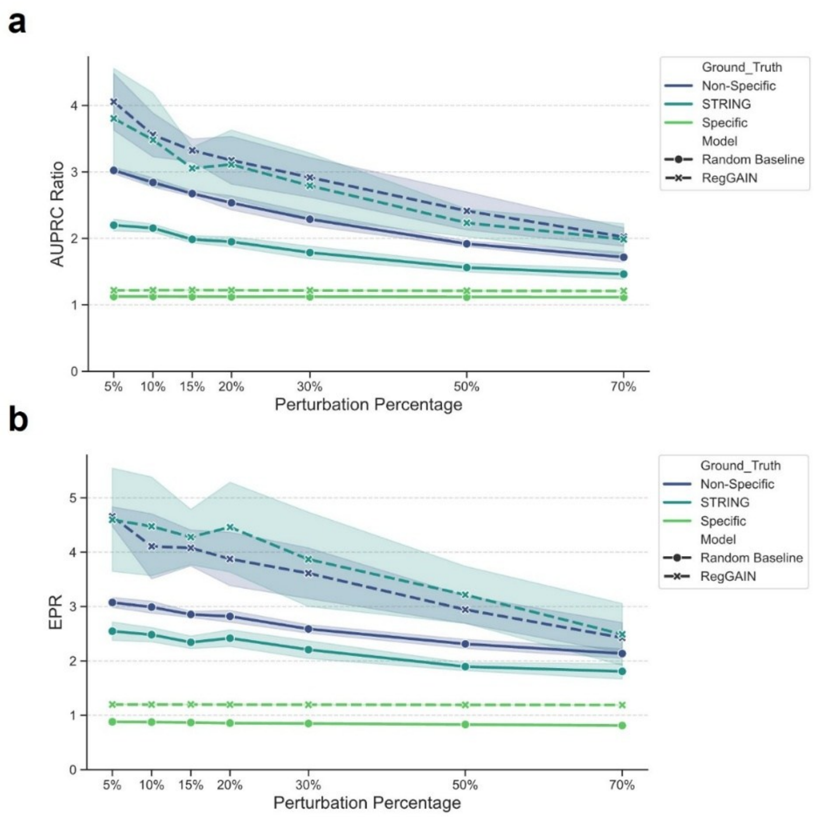

Figure S5. Robustness of RegGAIN against degree-preserving perturbations in the prior network. The plots show RegGAIN's performance, measured by (**a**) AUPRC ratio and (**b**) EPR, as the percentage of edges perturbed via a degree-preserving random swapping strategy is increased. This method rewires connections while keeping the in- and out-degree of each node constant. Dashed lines with 'x' markers represent the mean performance of RegGAIN, compared to a baseline model (solid lines with 'o' markers) that uses only the corrupted prior network.

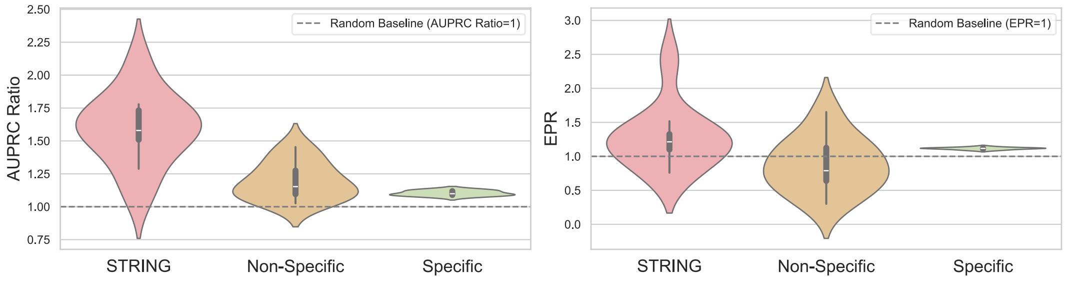

Figure S6. Violin plots show the distribution of AUPRC ratio and EPR across 10 replicates on three ground-truth networks. The prior network was an Erdős-Rényi (ER) random graph with the same number of nodes and edges as the original biological prior. The dashed line at 1.0 indicates the random baseline performance.

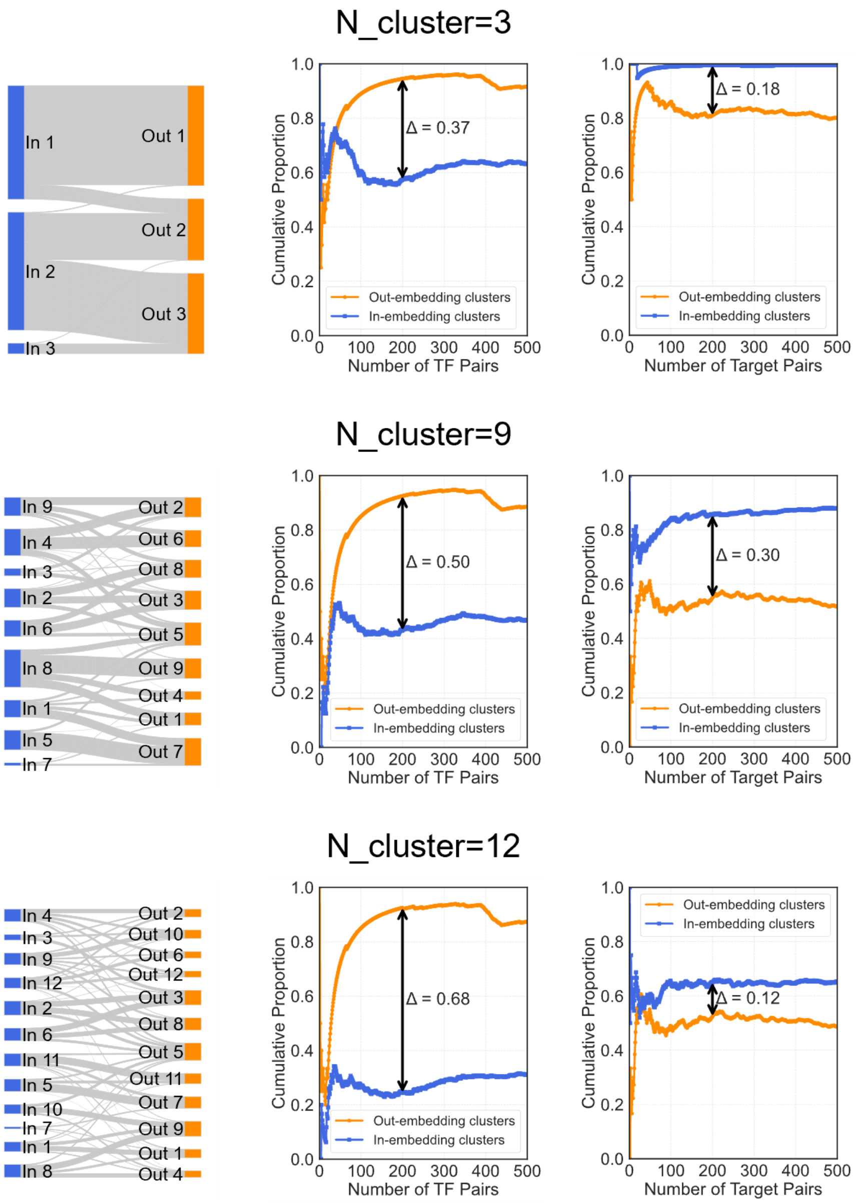

Figure S7. Sankey diagrams and line plots show the model's robustness to the number of k-means clusters (*N_cluster*). The Sankey diagrams show the mapping between in-embedding (blue) and out-embedding (orange) clusters. The line plots show the cumulative proportion of functionally related TF pairs and Target pairs that are grouped within the same cluster.

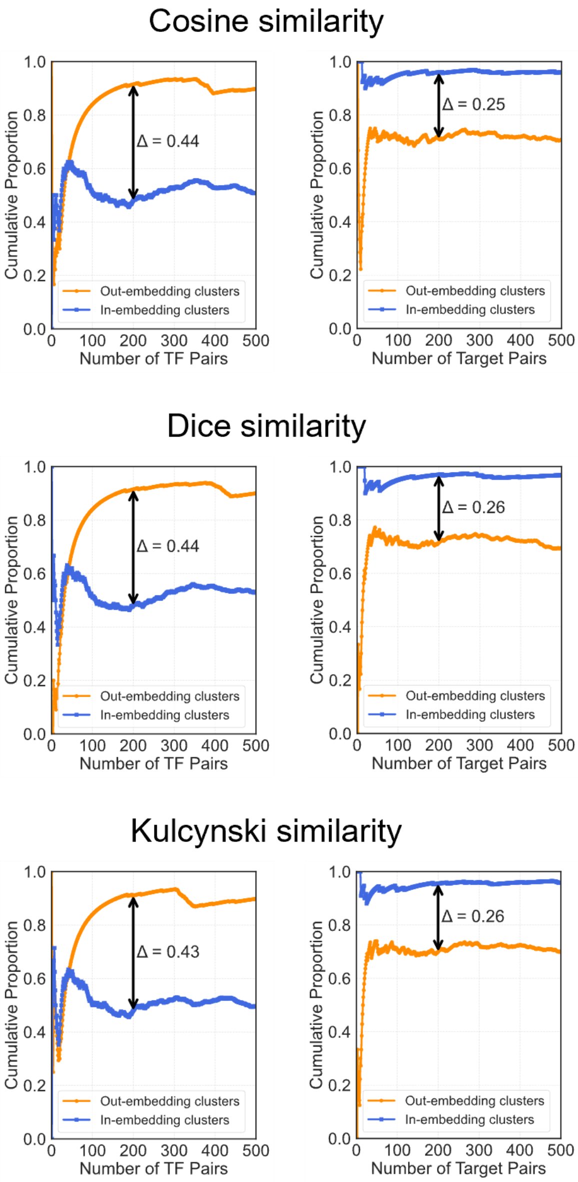

Figure S8. Line plots show the RegGAIN's robustness to the choice of similarity metric for defining TF relationships. The plots confirm that the distinct functional roles are maintained when using Cosine, Dice, and Kulczynski similarities.

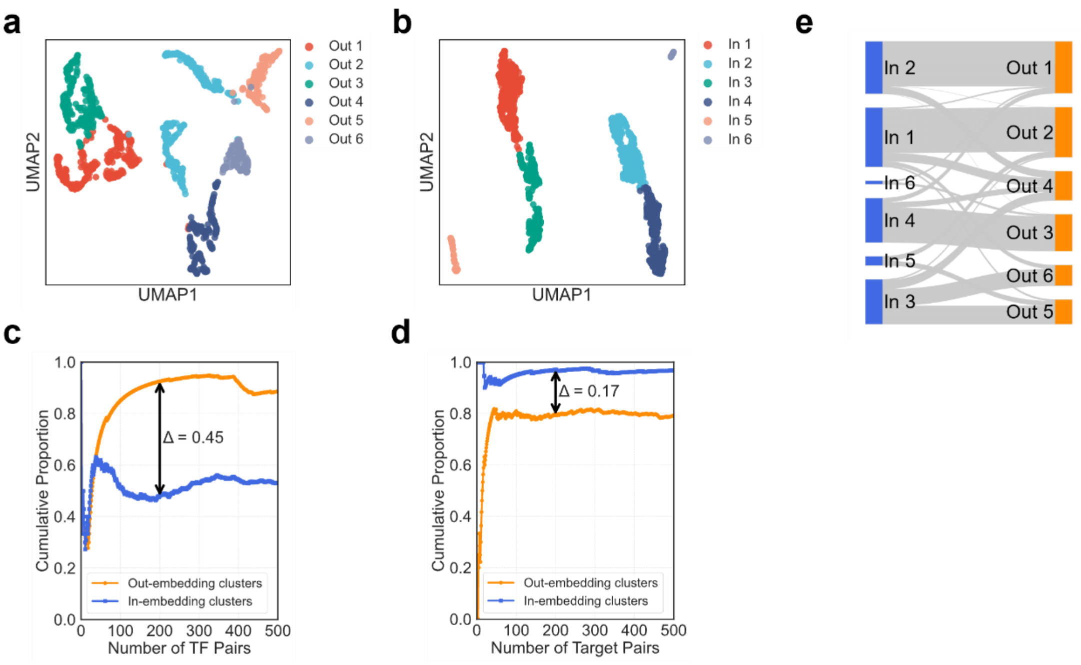

Figure S9. a, b, UMAP visualization of gene out-embeddings (**a**) and gene in-embeddings (**b**) for the hESC dataset, with clustering performed using the Leiden algorithm (resolution = 0.2). **c, d**, Line plots showing the cumulative proportion of the most similar TF pairs (**c**) and target gene pairs (**d**) that fall within the same cluster. **e**, Sankey diagram illustrating the correspondence between out-embedding clusters and in-embedding clusters.

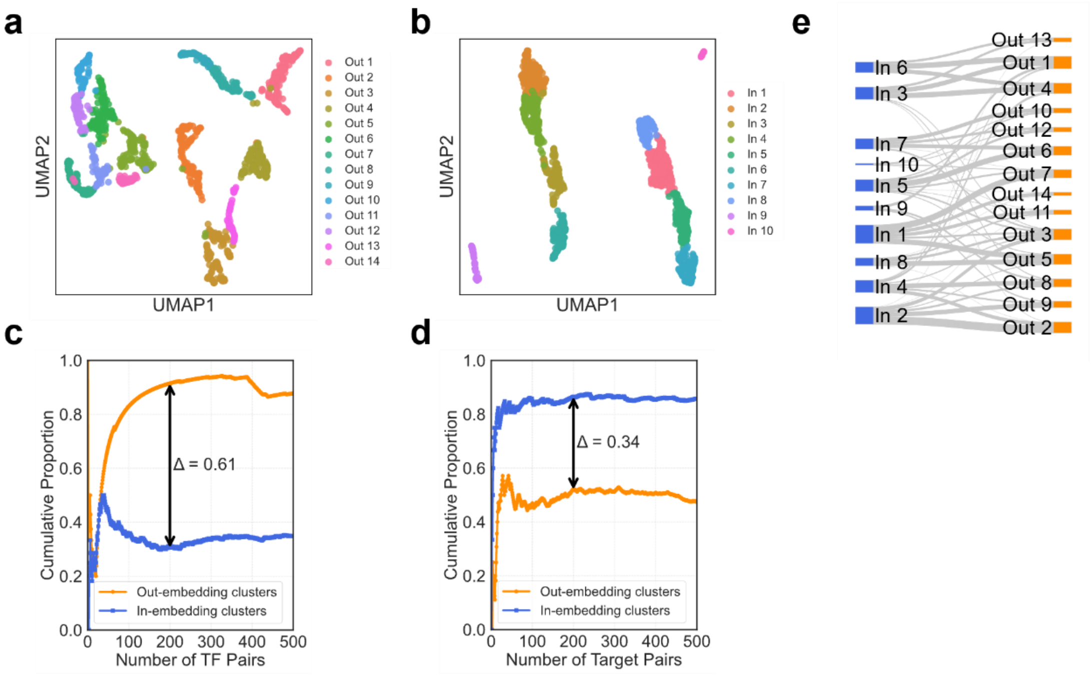

Figure S10. a, b, UMAP visualization of gene out-embeddings (**a**) and gene in-embeddings (**b**) for the hESC dataset, with clustering performed using the Leiden algorithm (resolution = 1). **c, d**, Line plots showing the cumulative proportion of the most similar TF pairs (**c**) and target gene pairs (**d**) that fall

within the same cluster. **e**, Sankey diagram illustrating the correspondence between out-embedding clusters and in-embedding clusters.

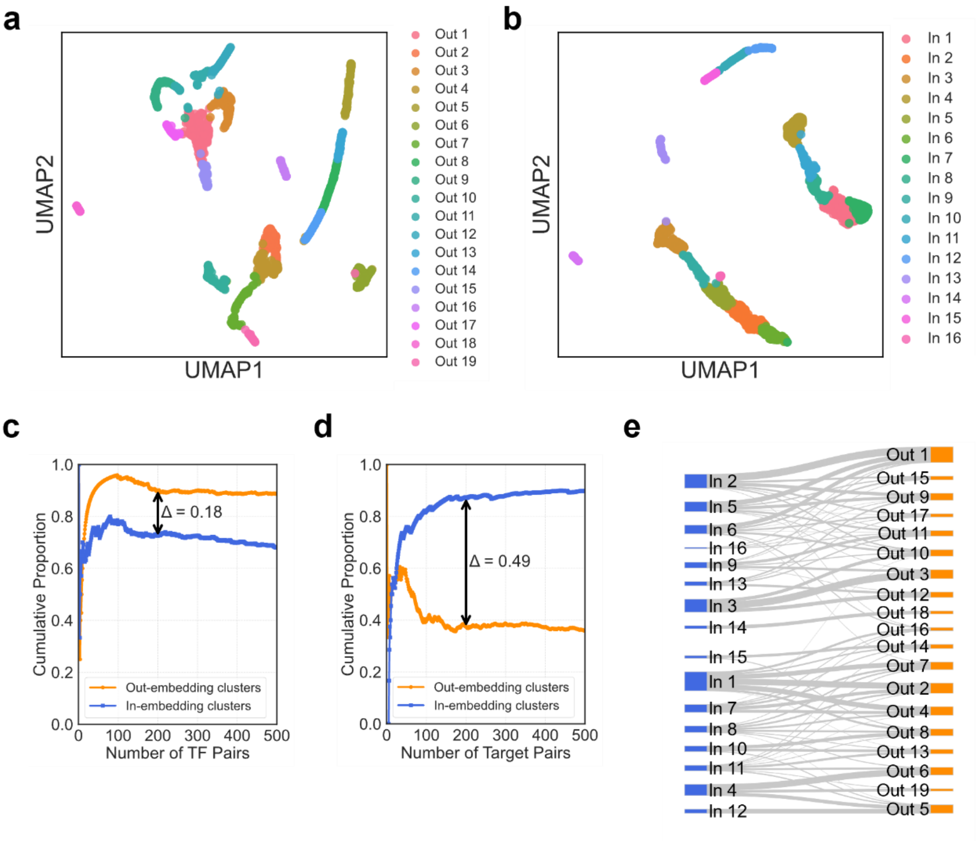

Figure S11. a, b, UMAP visualization of gene out-embeddings (**a**) and gene in-embeddings (**b**) for the mESC dataset, with clustering performed using the Leiden algorithm (resolution = 1). **c, d**, Line plots showing the cumulative proportion of the most similar TF pairs (**c**) and target gene pairs (**d**) that fall within the same cluster. **e**, Sankey diagram illustrating the correspondence between out-embedding clusters and in-embedding clusters.

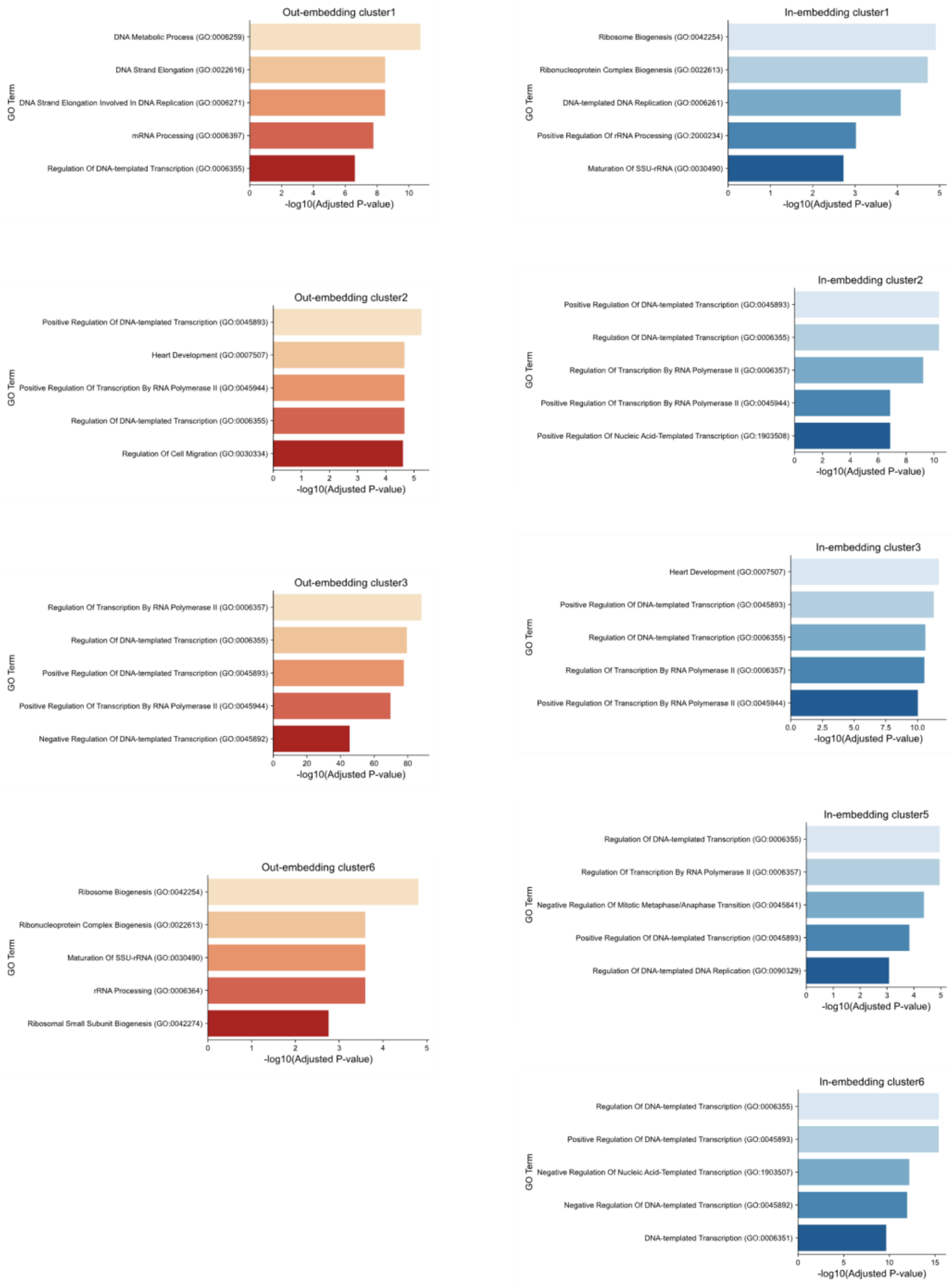

Figure S12. GO functional enrichment analysis (GO_Biological_Process_2023) was performed using the Enrichr tool for each out-embedding cluster (left) and in-embedding cluster (right). Each cluster's gene list underwent hypergeometric testing with FDR correction for multiple comparisons to identify significantly enriched terms. Clusters with insufficient gene numbers were excluded from the enrichment analysis.

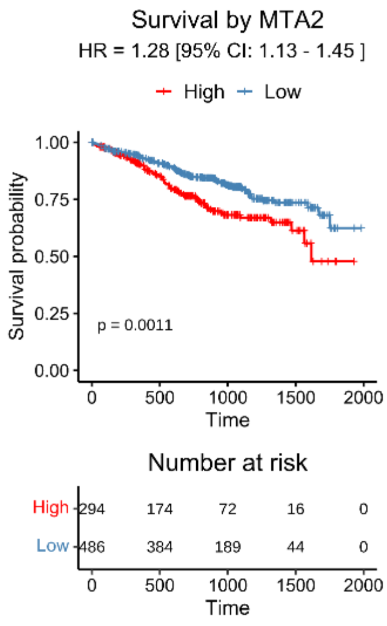

Figure S13. Kaplan–Meier survival curves illustrate the clinical relevance of the *MTA2* on the independent multiple myeloma cohort. Tick marks indicate censoring events. The statistical *p*-values were determined by the two-tailed log rank sum test.

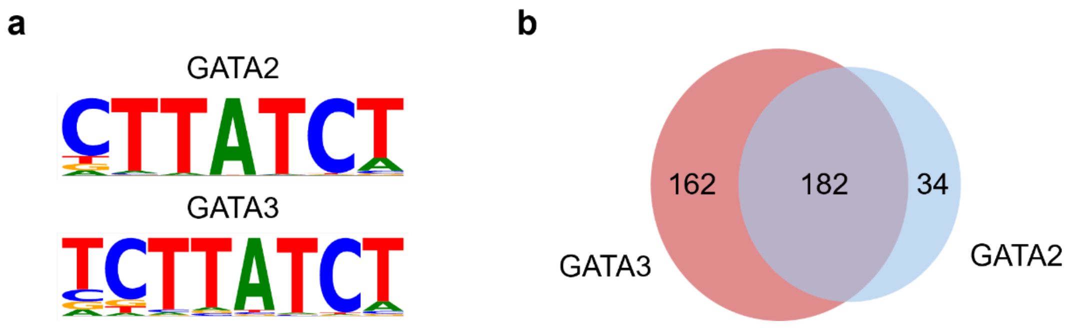

Figure S14. a, Sequence logos representing the DNA-binding motifs of *GATA2* and *GATA3*, showing the conserved nucleotide preferences for each TF. **b**, Venn diagram illustrating the overlap between predicted target genes of *GATA2* and *GATA3*, with numbers indicating unique and shared targets.

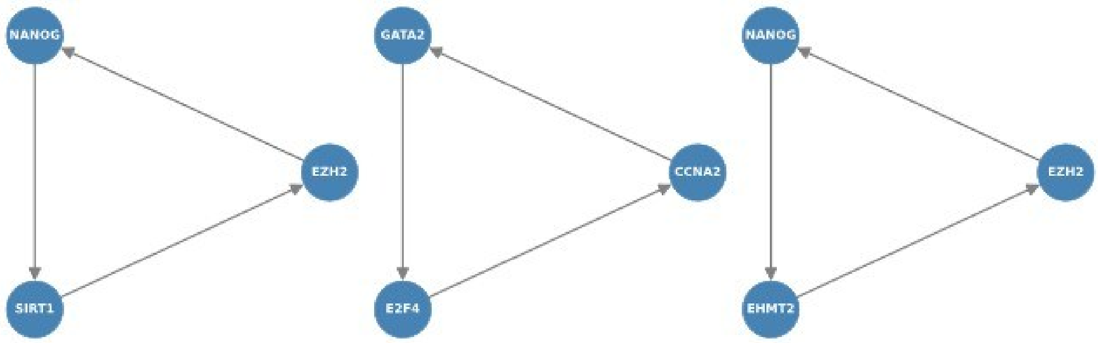

Figure S15. Visualization of representative feedback loops identified by RegGAIN. The plots show three distinct 3-gene feedback circuits discovered in the inferred hESC regulatory network.

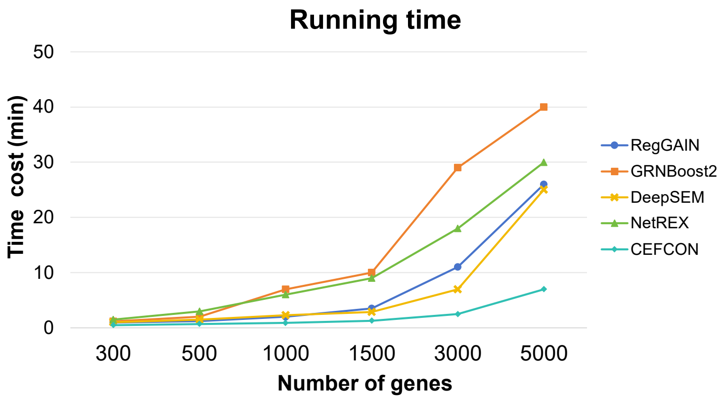

Figure S16. The running time comparison of RegGAIN and other baseline methods. Running time benchmarks were performed on a Linux server with Intel Xeon Platinum 8358 CPUs, 1 TB RAM, and NVIDIA A100 GPUs (40 GB memory).

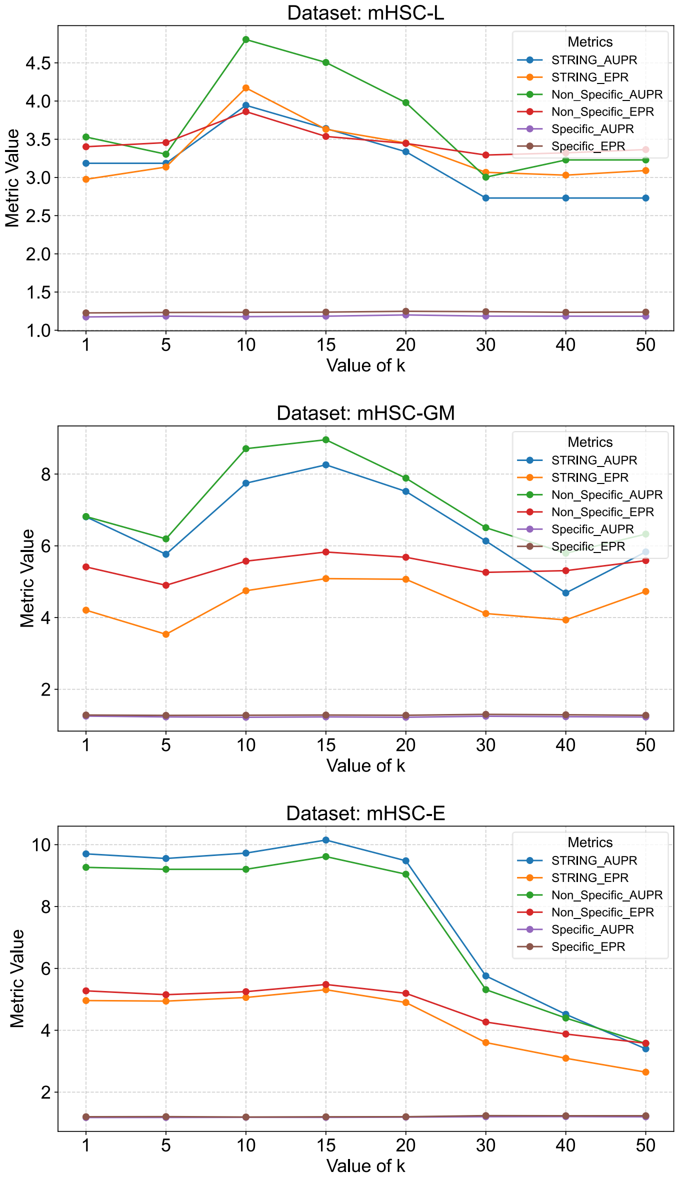

Figure S17. Sensitivity analysis for the selection of a percentile-based degree centrality threshold to define a special node type. The line plots show the RegGAIN's performance on three different mHSC datasets as a function of the percentile cutoff (*k*%) used to define a special node type. The x-axis represents the percentage of top-ranked nodes by degree centrality selected, while the y-axis shows the resulting performance measured by AUPRC ratio and EPR.

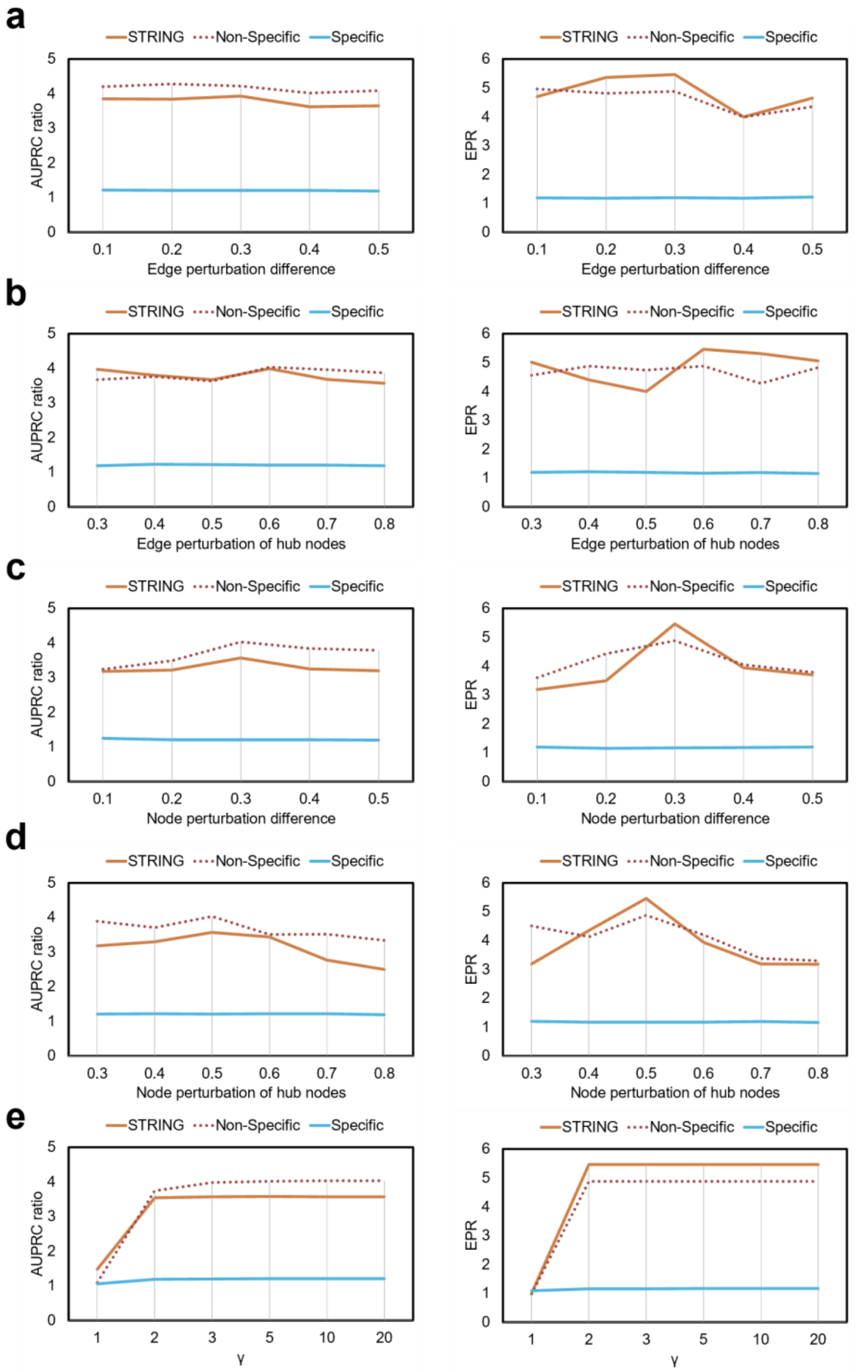

Figure S18. Performance of RegGAIN on the mHSC-L dataset under different parameter settings, evaluated using three ground-truth networks. **a**, Effect of varying the edge perturbation difference ($\alpha_2 - \alpha_1$) on AUPRC ratio (left) and EPR (right). **b**, Effect of the edge perturbation of hub nodes (α_2) in GRN prediction on AUPRC ratio (left) and EPR (right). **c**, Effect of varying the node perturbation difference ($\beta_2 - \beta_1$) on AUPRC ratio (left) and EPR (right). **d**, Effect of the node perturbation of hub nodes (β_2) in GRN prediction on AUPRC ratio (left) and EPR (right). **e**, Effect of the weighting parameter γ in GRN prediction on AUPRC ratio (left) and EPR (right).

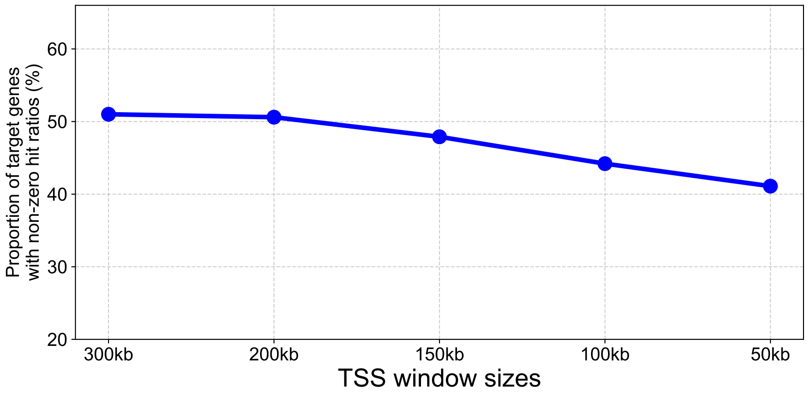

Figure S19. Line plot shows the robustness of the epigenetic validation to different genomic window sizes. The plot displays the proportion of target genes with non-zero hit ratios as a function of the genomic window size centered on the TSS.

3. Supplementary Tables

Table S1. Detailed information of the seven scRNA-seq benchmark datasets.

(Cell×Gene)	TFs+500 HVGs	TFs+1,000 HVGs
hESC	758×910	758×1,410
hHEP	425×948	425×1,448
mDC	383×821	383×1,321
mESC	421×1,120	421×1,620
mHSC-E	1,071×704	1,071×1,204
mHSC-GM	889×632	889×1,132
mHSC-L	847×560	847×693

Table S2. Detailed information of the three kinds of ground-truth networks of the seven scRNA-seq datasets.

	STRING		Non-Specific		Cell-type Specific	
	TF+500	TF+1,000	TF+500	TF+1,000	TF+500	TF+1,000
hESC	4,257	5,149	3,441	4,617	4,545	7,084
hHEP	7,523	9,003	4,129	5,351	9,939	15,558
mDC	4,815	5,898	3,067	3,918	756	1,193
mESC	7,762	8,479	6,893	8,030	29,613	42,795
mHSC-E	1,371	1,826	1,425	1,960	11,557	21,975
mHSC-GM	748	1,311	743	1,358	7,364	14,135
mHSC-L	317	154	279	317	4,398	5,180

Table S3. Detailed information of edge counts in prior gene interaction networks (NicheNet) used as inputs for RegGAIN across seven scRNA-seq datasets.

Edge counts in prior network	TFs+500 HVGs	TFs+1,000 HVGs
hESC	63,565	107,769
hHEP	98,373	163,288
mDC	97,606	179,757
mESC	144,032	219,870
mHSC-E	38,802	86,888
mHSC-GM	21,881	66,468
mHSC-L	11,389	14,601

Table S4. Detailed information on the three types of ground-truth networks and their overlap with the prior gene interaction network (NicheNet) across the seven scRNA-seq datasets. For each dataset, the top and bottom rows show the total number of ground-truth edges and their overlap with the prior network.

Datasets	STRING		Non-Specific		Cell-type Specific	
	TF+500	TF+1,000	TF+500	TF+1,000	TF+500	TF+1,000
hESC	4,257	5,149	3,441	4,617	4,545	7,084
	3,554	3,997	3,369	4,461	3,086	4,611
hHEP	7,523	9,003	4,129	5,351	9,939	15,558
	6,349	6,999	4,059	5,243	8,814	13,589
mDC	4,815	5,898	3,067	3,918	756	1,193
	4,119	4,772	2,738	3,495	494	745
mESC	7,762	8,479	6,893	8,030	29,613	42,795
	6,692	6,905	6,774	7,886	15,444	19,892
mHSC-E	1,371	1,826	1,425	1,960	11,557	21,975
	1,085	1,315	1,407	1,937	6,479	11,867
mHSC-GM	748	1,311	743	1,358	7,364	14,135
	559	891	735	1,340	4,050	7,946
mHSC-L	137	154	279	317	4,398	5,180
	110	115	274	308	2,098	2,353

Table S5. Detailed information of the input prior gene interaction networks.

Networks	Species	Source genes	Target genes	Total genes	Edges
NicheNet	Human	18,569	25,332	25,345	5,290,993
	Mouse	17,455	18,579	18,759	5,029,532
Pathway Commons	Human	16,688	18,689	19,087	1,105,240
	Mouse	15,832	17,590	17,834	1,098,689
InBioMap	Human	14,462	16,075	17,430	609,015
	Mouse	13,742	15,350	16,438	554,357
Harmonizome	Human	4,319	26,381	26,780	2,985,645
	Mouse	3,836	18,002	18,002	2,446,888
Omnipath	Human	6,182	7,435	8,725	86,248
	Mouse	6,154	7,524	8,726	79,693

Table S6. RegGAIN's AUPRC ratio performance and overall rank on different prior gene networks using TF+1,000 HVGs.

AUPRC ratio		NicheNet	NicheNet_D	Pathway Common	inbiomap	Harmonize	Omnipath
STRING	hESC	6.76	5.41	7.31	7.08	1.32	4.34
	hHEP	7.55	4.77	7.24	7.71	1.2	3.98
	mDC	4.64	3.44	4.34	5.16	1.03	3.48
	mESC	5.71	6.02	5.46	5.21	1.32	4.85
	mHSC-E	5.38	3.63	5.13	4.82	1.12	3.33
	mHSC-GM	4.40	3.73	3.74	3.56	1.15	2.66
	mHSC-L	3.41	2.87	3.43	2.86	1.56	4.31
Overall Rank		1	4	2	3	6	5
Non-Specific	hESC	5.75	8.09	3.99	2.62	3.33	3.44
	hHEP	4.58	6.81	4.25	2.73	3.07	3.15
	mDC	3.76	4.81	2.91	2.73	1.92	3.17
	mESC	6.22	9.9	3.95	4.52	2.25	4.05
	mHSC-E	5.95	5.12	4.44	3.04	1.77	2.28
	mHSC-GM	5.54	6.98	3.15	2.83	1.89	1.97
	mHSC-L	3.69	4.45	2.38	1.73	1.56	2.00
Overall Rank		2	1	3	4	6	5
Cell-type-Specific	hESC	1.29	1.31	0.98	1.01	1.37	1.06
	hHEP	1.28	1.35	1	0.95	1.39	0.98
	mDC	1.13	1.12	1.04	1.1	1.13	1.08
	mESC	1.25	1.32	1.08	1.05	1.32	1.03
	mHSC-E	1.19	1.25	1.08	1.06	1.24	1.04
	mHSC-GM	1.27	1.31	1.14	1.12	1.29	1.14
	mHSC-L	1.24	1.23	1.09	1.07	1.24	1.08
Overall Rank		3	2	4	6	1	4

Table S7. RegGAIN's EPR performance and overall rank on different prior gene networks using TF+1,000 HVGs.

EPR		NicheNet	NicheNet_D	Pathway Common	inbiomap	Harmonize	Omnipath
STRING	hESC	10.25	7.83	14.62	12.83	1.77	4.55
	hHEP	11.91	7.24	13.19	12.49	1.27	4.21
	mDC	6.68	4.4	8.93	10.94	1.02	4.37
	mESC	8.41	8.77	11.52	12.33	1.63	6.28
	mHSC-E	9.64	4.55	9.79	8.89	1.20	3.88
	mHSC-GM	7.30	5	6.41	5.34	1.11	2.76
	mHSC-L	4.37	3.91	6.59	3.68	2.09	3.77
Overall Rank		3	4	1	2	6	5
Non-Specific	hESC	5.14	9.81	11.05	8.18	5.08	5.96
	hHEP	3.25	7.87	10.42	10.47	3.94	6.05
	mDC	4.29	6.47	7.51	8.91	2.68	6.35
	mESC	7.16	13.9	10.26	15	3.24	7.34
	mHSC-E	9.12	5.69	10.81	7.76	2.25	3.46
	mHSC-GM	7.62	7.69	8.19	5.01	2.55	2.49
	mHSC-L	4.05	4.98	4.12	2.53	1.99	1.78
Overall Rank		4	3	1	2	6	5
Cell-type-Specific	hESC	1.34	1.36	0.96	1.01	1.44	1.03
	hHEP	1.34	1.39	0.97	0.93	1.4	0.98
	mDC	1.07	1.16	1.13	1.13	1.15	1.15
	mESC	1.38	1.39	1.08	1.06	1.37	1.04
	mHSC-E	1.15	1.17	1.05	1.04	1.17	1.03
	mHSC-GM	1.21	1.22	1.10	1.08	1.19	1.12
	mHSC-L	1.19	1.28	1.05	1.05	1.16	1.08
Overall Rank		3	1	5	6	2	4

References

[1] A. Pratapa, A. P. Jalihal, J. N. Law, A. Bharadwaj, and T. M. Murali, "Benchmarking algorithms for gene regulatory network inference from single-cell transcriptomic data," *Nat. Methods*, vol. 17, no. 2, pp. 147–154, Feb. 2020, doi: 10.1038/s41592-019-0690-6.

[2] T. Moerman *et al.*, "GRNBoost2 and Arboreto: efficient and scalable inference of gene regulatory networks," *Bioinformatics*, vol. 35, no. 12, pp. 2159–2161, June 2019, doi: 10.1093/bioinformatics/bty916.

[3] H. Shu *et al.*, "Modeling gene regulatory networks using neural network architectures," *Nat. Comput. Sci.*, vol. 1, no. 7, pp. 491–501, July 2021, doi: 10.1038/s43588-021-00099-8.

[4] P. Li, L. Li, J. Nan, J. Chen, J. Sun, and Y. Cao, "KEGNI: knowledge graph enhanced framework for gene regulatory network inference," *Genome Biol.*, vol. 26, no. 1, p. 294, Sept. 2025, doi: 10.1186/s13059-025-03780-7.

[5] Y. Wang, D.-Y. Cho, H. Lee, J. Fear, B. Oliver, and T. M. Przytycka, "Reprogramming of regulatory network using expression uncovers sex-specific gene regulation in Drosophila," *Nat. Commun.*, vol. 9, no. 1, Oct. 2018, doi: 10.1038/s41467-018-06382-z.

[6] P. Wang *et al.*, "Deciphering driver regulators of cell fate decisions from single-cell transcriptomics data with CEFCON," *Nat. Commun.*, vol. 14, no. 1, p. 8459, Dec. 2023, doi: 10.1038/s41467-023-44103-3.

[7] R. Browaeys, W. Saelens, and Y. Saeys, "NicheNet: modeling intercellular communication by linking ligands to target genes," *Nat. Methods*, vol. 17, no. 2, pp. 159–162, Feb. 2020, doi: 10.1038/s41592-019-0667-5.

[8] I. Rodchenkov *et al.*, "Pathway Commons 2019 Update: integration, analysis and exploration of pathway data," *Nucleic Acids Res.*, Oct. 2019, doi: 10.1093/nar/gkz946.

[9] T. Li *et al.*, "A scored human protein-protein interaction network to catalyze genomic interpretation," *Nat. Methods*, vol. 14, no. 1, pp. 61–64, Jan. 2017, doi: 10.1038/nmeth.4083.

[10] A. D. Rouillard *et al.*, "The harmonizome: a collection of processed datasets gathered to serve and mine knowledge about genes and proteins", Accessed: July 07, 2025. [Online]. Available: https://dx.doi.org/10.1093/database/baw100

[11] D. Türei, T. Korcsmáros, and J. Saez-Rodriguez, "OmniPath: guidelines and gateway for literature-curated signaling pathway resources," *Nat. Methods*, vol. 13, no. 12, pp. 966–967, Dec. 2016, doi: 10.1038/nmeth.4077.
